# Technology Adoption and Early Network Infrastructure Provision in the Market for Electric Vehicles

**DOI:** 10.1007/s10640-022-00703-z

**Published:** 2022-07-07

**Authors:** Jeremy van Dijk, Nathan Delacrétaz, Bruno Lanz

**Affiliations:** 1 Institute of Economic Research, University of Neuchâtel, Rue A.-L. Breguet 2, 2000 Neuchâtel, Switzerland; 2 Center for Integrative Risk Management and Economics, ETH Zürich, Zürich, Switzerland; 3 Center for Energy and Environmental Policy Research and Joint Program on the Science and Policy of Global Change, Massachusetts Institute of Technology, Cambridge, USA

**Keywords:** Technology adoption, Network externality, Electric vehicles, Charging infrastructure, Two-sided markets, Behavioral bias, Range anxiety, Environmental policy

## Abstract

We document non-linear stock effects in the relationship linking emerging technology adoption and network infrastructure increments. We exploit 2010–2017 data covering nascent to mature electric vehicle (EV) markets across 422 Norwegian municipalities together with two complementary identification strategies: control function regressions of EV sales on flexible polynomials in the stock of charging stations and charging points, and synthetic control methods to quantify the impact of initial infrastructure provision in municipalities that previously had none. Our results are consistent with indirect network effects and the behavioral bias called “range anxiety,” and support policies targeting early infrastructure provision to incentivize EV adoption.

## Introduction

The demand for personal mobility is associated with significant local and global externalities, and many countries consider electrification as the future of on-road transportation.^1^ Even in the presence of externality-correcting taxes, however, indirect network effects hamper decisions to purchase an electric vehicle (EV) at the individual level (Greaker and Midttomme 2016). In particular, the benefit of EV adoption depends on the size of charging infrastructure, whereas providers of charging stations will not invest in infrastructure provision when the base of EVs in circulation is small. In the presence of unpriced benefits to consumers (e.g. lower search costs), the private deployment of network infrastructure is likely suboptimal (Farrell and Saloner 1986; Katz and Shapiro 1986; Cabral 2011). In turn, policies supporting the early provision of public charging infrastructure can alleviate a chicken and egg dilemma between EV consumers and charging station providers.

**Fig. 1:**
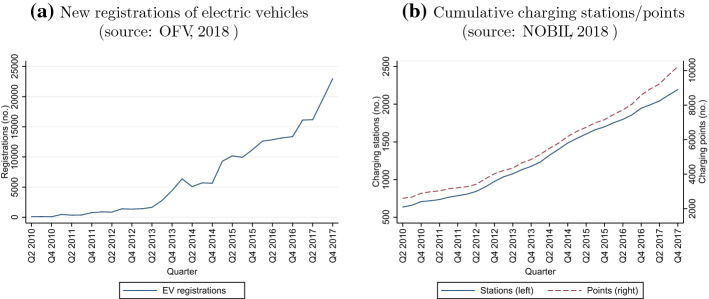
Electric vehicle registrations and charging stations/points in Norway, 2010–2017

In this setting, the objective of this paper is to provide novel evidence about how increments to charging infrastructure affect EV adoption decisions, and study how consumers respond to charger installations at early and developed market stages. We employ data for all 422 Norwegian municipalities from 2010 (the first year of comprehensive charger data availability) to 2017, with quarterly information on EV registrations (both battery-only electric vehicles—BEV—and plug-in hybrid vehicles—PHEV) by make and model, and the number of available charging stations, together with the number of charging points within these. Figure 1a illustrates how registrations of new EVs increased from around 90 in Q2 2010 to around 23,000 in Q4 2017, the latter representing 49% of all new car registrations (OFV 2018), the world’s highest rate of EV use (International Energy Agency 2019). Over the same period, the number of charging stations increased from around 640 in Q2 2010 to 2194 by the end of 2017 (Fig. 1b). Charging points follow a similar trend, rising from around 2,600 to 10,240 over the period. Note that EVs can sometimes be recharged at home, however potential adopters still derive utility from the availability of *public* charging infrastructure. Particularly, Norwegian geographical specificities make a public charging network important (eg. large distances, cold weather, mountainous terrain—see Springel (2021) for a discussion). This issue is especially important for battery-only EVs (BEVs), but plug-in hybrids (PHEVs) can also benefit from public charging stations once they run out of electricity.

Norway has implemented a range of incentive schemes to promote EVs, including subsidies for charging infrastructure, financial incentives such as exemptions from registration tax and VAT, the free use of toll roads, public parking spaces and bus lanes, and discounted ferry tickets. The Bjerkan et al. (2016) survey, for example, showed that these incentives have varying importance to consumers and differing impacts on uptake, but financial incentives are by far the most critical for purchasing decisions.^2^ For a small subset of the population, however, non-financial incentives, particularly free toll road and bus lane use, were ‘critically important’ for their EV purchase decision. We emphasize that these policies were mostly implemented nationally before 2010 (Norwegian EV Association 2021; Fevang t al. 2020), have limited within-municipality temporal variation, and are controlled for in our estimation strategy. Our objective is to isolate exogenous variation in charging infrastructure and quantify its impact on EV purchase.

We use two complementary strategies to identify the impact of charging infrastructure on EV adoption from the emergence of the market in 2010 to a more mature market in 2017. First, we regress the log of new EV registrations on the log of charging stations available in a given municipality-quarter, and thereby estimate the elasticity of EV purchases with respect to incremental charging infrastructure. The primary issue with this analysis, however, is endogeneity in the municipality-level availability of charging infrastructure (Li et al. 2017). In particular, demand for EVs and the availability of charging infrastructure are potentially jointly affected by unobserved factors such as environmental preferences and associated government policies (e.g. subsidies for local charging infrastructure). Moreover, indirect network effects imply a reverse causality problem whereby greater EV registrations lead to more charger installations, for example through higher expected financial returns.

To isolate the impact of incremental charging infrastructure on EV adoption, we follow Li et al. (2017) and construct a Bartik (1991) instrument based on the stock of public parking spaces available in each municipality and the nation-wide trend of charger installations.^3^ In this context, identification rests on two assumptions: (i) more abundant parking space isolates plausibly exogenous variation in the opportunity to supply charging infrastructure, and (ii) municipalities with more parking space are more likely to respond to a nation-wide trend in EV adoption.^4^ Importantly, these assumptions are conditional on a set of control variables capturing differential changes in prices and income, among other things, as well as quarter fixed effects (capturing national technology trends and policy incentives for EVs), and municipality-model fixed effects (controlling for time invariant EV attributes and within-municipality preferences, as well as municipality characteristics, such as proximity to workplaces and urban centres, commuting routes, and toll road, bus lane and ferry prevalence). This instrumental variable (IV) approach limits any potential omitted variable bias.

Based on this, the first contribution of this paper is to exploit the development of the EV market in Norway to investigate how the pre-existing stock of installed charging stations affects the charger-elasticity of EV demand. We use a set of control function (CF) regressions (Wooldridge 2015) in which residuals from the first stage are included in the second stage, allowing us to estimate flexible polynomial specifications in the size of the charging infrastructure.^5^ Our results show that charger-elasticity estimates increase with the stock of charging stations, which suggests that incremental charger installations are subject to increasing returns from network externalities. We further show that the largest impact of incremental charging infrastructure occurs when there is little to no pre-existing charger network. As discussed in Meunier and Ponssard (2020), this is consistent with declining marginal benefits associated with charging infrastructure as the size of the network grows (e.g. through declining disutility associated with locating and reaching a charging point). From a policy perspective, this suggests that subsidizing early infrastructure provision in small EV markets can mitigate the associated inefficiencies and therefore complement other instruments tackling transport externalities (e.g. a carbon tax).

Quantitatively, we estimate that a 10% increase in charging stations causes a rise in EV registrations by around 2.2% at the mean of our sample.^6^ We further provide suggestive evidence that consumers respond differently to the provision of charging *points*, with a corresponding estimate of 1.2%. A higher elasticity for the provision of stations versus points is consistent with existing empirical evidence documenting a behavioral bias called “range anxiety”, whereby drivers tend to systematically over-estimate their required driving range.^7^ See for example DeShazo et al. (2017) and Dimitropoulos et al. (2016). This behavioral effect magnifies the network externality problem, and suggests that expanding the network of infrastructure with charging stations with a single or few charging point(s) delivers the greatest benefits to consumers.

The second empirical strategy is geared towards the role of initial infrastructure provision. We focus on a subset of 64 Norwegian municipalities with a base of zero charging stations in 2010 and for which we observe either just one station being installed (one-station group) or multiple stations installed within a window of 4 consecutive quarters (multi-station group). To quantify the impact of this one-off infrastructure provision on EV registrations, we employ the synthetic control method (SCM—Abadie and Gardeazabal 2003; Abadie et al. 2010).^8^ In this approach, a synthetic municipality is constructed by giving weights to all those in a set of potential control units (the donor pool), which we take to be all municipalities that never installed any charging stations over the entire observation period. The weights attributed to each municipality in the donor pool are selected so as to minimize pre-treatment differences in cumulative EV sales between a given treated unit and the synthetic municipality. For this purpose, we implement the ridge-augmented SCM (Ben-Michael et al. 2018), which adds a bias-correction term to the original SCM weights and allows for the use of negative weights in the construction of the synthetic control unit (see also Abadie and Imbens 2011).

Building on an absence of difference in EV registrations for pairs of treated and synthetic municipalities during the pre-treatment period, the trajectory for the synthetic municipality can be interpreted as a counterfactual trajectory for EV adoption in the absence of treatment. Consequently, a comparison of the treated municipalities and their respective synthetic municipalities quantifies the impact of initial infrastructure provision on cumulative EV purchases. Overall, our results suggest a positive impact of the first charging stations. One year after the installation the cumulative EV sales in treated municipalities increases on average by 5.4% for one-station group and 8.0% for multi-station group relative to control. The average treatment effect increases with time, and 2 years post-treatment we estimate 21.7 and 46.2% increases in the one-station and multi-station groups respectively. These results confirm large (unpriced) consumer benefits associated with early infrastructure provision, so that policy intervention in nascent markets can significantly contribute to initiate adoption dynamics.

These results contribute to a broad literature on indirect network effects and two-sided markets in relation to early technology adoption (see Caillaud and Jullien 2003; Armstrong 2006; Rochet and Tirole 2006). For example, Gandal et al. (2000) studies the adoption of CDs and how this depends on and affects the diffusion of CD player hardware, so that both sides of the market await developments in the other before making a commitment. Rysman (2004) demonstrates a positive network effect in the two-sided Yellow Pages market, and Rochet and Tirole (2002) analyze the interaction between payment card users (consumers) and merchants’ acceptance of such cards. Lee (2013) investigates the feedback between consumer demand for video game hardware and software, and software demand for various hardware platforms, demonstrating the negative impact of incompatibility. In our context, these network effects hinder the effect of policies targeting externalities associated with mobility, and therefore call for a policy intervention.

Our work also contributes to a growing literature focusing on the adoption of EVs.^9^ In particular, our work is closely related to Li et al. (2017), who study the early development of the U.S. market for EVs based on 2011 to 2013 data for 353 metropolitical statistical areas (MSA) with significant EV sales. They employ a Bartik-style instrument based on the number of local supermarkets to generate exogenous variation in the provision of charging stations, which also uses an assumption that more abundant parking areas facilitate the installation of EV chargers without affecting the trade-off between EVs and standard vehicles. They report an elasticity of around 0.8, which is significantly larger than our central estimate (0.22). Our results suggest, however, that part of this difference can be attributed to the size of the stock of charging infrastructure in MSAs considered in their analysis: 22.13 in Li et al. (2017), and only 3.09 in our data. Using our polynomial specification, we find that the elasticity corresponding to a stock of stations of 22 in our data is 0.54, which illustrates the importance of studying early infrastructure provision in the design of policies supporting EV adoption.

Related evidence focuses on the role of policy incentives for the adoption of EVs. For example, Clinton and Steinberg (2019) uses 2011 to 2015 data for the U.S. to quantify the impact of direct financial incentives in Texas and Massachusetts on EV adoption.^10^ Using both panel data and SCM, they show that subsidies increase adoption, although they suggest that the net welfare effect of direct EV subsidies is negative. Similarly, Springel (2021) uses 2010 to 2015 data for 19 Norwegian counties to study subsidies for EVs and charging stations.^11^ She estimates a structural demand model for EVs, showing that subsidizing charging stations is more efficient than directly subsidizing EVs. Relative to these two studies, we provide a first set of empirical results suggesting that indirect network effects are large when the stock of charging stations is small, which provides novel insights for optimal policy targeting charging infrastructure provision in nascent EV markets (Meunier and Ponssard 2020).

Finally, our research is related to the non-monetary and psychological barriers to adoption of new energy technologies demonstrated by Fowlie et al. (2015). Jaffe and Stavins (1994) argue that a lack of uptake of energy efficient technologies is due to factors such as incomplete information and unobserved costs, while Heutel and Muehlegger (2015) shows that consumer learning about the practical use and attributes of new technologies increases adoption. Other papers demonstrate the effect of community and personal environmental preferences on the adoption of traditional hybrid vehicles (Kahn 2007; Kahn and Vaughn 2009), for which we account in our analysis.

One question we do not directly address is different charger speeds (fast vs. slow) and their relative benefits in varied use-cases. In theory, slow chargers may be better suited to urban areas and locals charging as a supplement to or replacement of home-charging. Fast chargers could be more beneficial on long-distance driving routes, leisure destinations, and therefore, potentially, rural areas. As Greaker (2021) discusses, fast charger availability has been given as an important factor for EV adoption, particularly as an enabler of long-distance and leisure trips. Their theoretical model finds that fast charger standardisation and infrastructure roll-out would increase EV purchases and consumer welfare. Our paper somewhat abstracts from these differences, using overall charger numbers. However, on average, 85% of charging stations in our data are slow chargers. This, along with our analysis of within-municipality network and EV purchase variations, gives us confidence that we are analysing the local effects of nearby charger installations on EV adoption.

This paper proceeds as follows. Section 2 outlines our empirical strategy, first by providing our data and laying out summary statistics, and second by detailing our panel data and SCM approaches. Section 3 then reports our empirical results. Finally, Sect. 4 provides concluding comments.

## Empirical Strategy

In this section we first give a summary of our data, and then present our two complementary empirical approaches to identify the impact of charging infrastructure on EV demand.

### Data Overview

Our dataset covers all of Norway’s 422 municipalities for each quarter from Q3 2010 to Q4 2017 ($$\hbox {T}=30$$). The data includes the quantity of newly registered EVs by car model, month and municipality, and the prices for each car (OFV 2018). Car models here refer to the broadest classification thereof (e.g. Tesla Model S or Nissan Leaf). We obtain data on every publicly accessible EV charging station across Norway from the Norwegian Charging Station Database (NOBIL 2018), including its location, opening date and number of charging points.^12^$$^{,}$$^13^ Other variables capturing municipality-level characteristics originate from Statistics Norway (SSB 2018).

Table 1 summarizes our data. The average quantity of each EV model sold per quarter in each municipality is 0.56, and the total number of EVs sold of all models per municipality per quarter is over 16 on average. Note that, since EV models enter and exit the Norwegian car market over the period considered, we have an unbalanced panel. In 2010 there are only 4 models available, and this rose progressively to reach 50 in 2017.

**Table 1:** Descriptive statistics for all 422 Norwegian municipalities

	Mean	Std. Dev.	Min	Max
New EVs per model	0.56	5.44	0	528
Total of new EVs	16.07	93.54	0	3815
EV models available	28.93	14.64	4	50
Charging stations	3.09	14.36	0	376
Charging points	13.46	78.77	0	2331
Points per station	3.63	2.48	1.00	40.33
Parking spaces	570.06	1472.56	0	19,719
Car price	547,575.60	395,827.50	124,108.30	2,027,016.00
Mean household income	385,606.10	40,471.95	285,091.80	841,848.80
Hybrids 2008	10.11	46.09	0	736
Population	121,000.97	37,064.02	196	672,062
Detached houses	85.52	12.37	14.61	100

Data sources are OFV (2018), NOBIL (2018), and SSB (2018). Car price and mean municipal household income are measured in 2015 Norwegian kroner (NOK), with 1 USD approx. 8 NOK in 2015. Detached houses is measured as the percentage of all households that are detached or duplex

The number of charging stations available per municipality and quarter ranges between 0 and 376, with an average of 3.09. These values indicate large differences in charging infrastructure between municipalities and over time. Moreover, while the average number of charging points available is over 13, many charging stations only provide 1 or 2 points. Although the average municipal-level number of points per station goes up to 40 points.

We further use the number of parking places per municipality in 2017 as part of our instrument, which averages 570 and also has a large range. As additional control variables we use the car price, household income, the number of hybrid vehicles per municipality in 2008, population size, the proportion of households in a municipality that are detached houses or duplexes (as a proxy for level of urbanisation), and, separately, the categorical degree of urbanisation (urban/city, suburban/town, rural). The proportion of municipalities classified as urban is 2.8%, while 22.3% are sub-urban, and 74.9% rural. The number of traditional (ICE-) hybrid vehicles is used as an indicator of municipal green preferences before the mass-introduction of EVs, and willingness to buy new, green car technologies.

One remarkable feature of the data is that, despite the relatively large market share of EVs, there are still many Norwegian municipalities that have either no or very few charging station installations over our observation period. We exploit this feature of the data with a SCM strategy. First, 110 municipalities had zero charging stations over the entire period (donor municipalities). Second, we observe 47 municipalities that installed a single charging station in 1 quarter between Q1 2011 and Q1 2017, with no installations before or after (one-station municipalities). Third, we additionally observe 17 municipalities that installed multiple stations over a period of up to 4 consecutive quarters, however that had zero stations prior to Q1 2011 and no more after their 4-quarter installation period (multi-station municipalities). In this group, between 2 and 13 stations were installed over the installation period, with an average of 2.94.

**Table 2:** Descriptive statistics for municipalities included in the synthetic control analysis

	Mean	Median	Std. Dev.	Min	Max
*One-station municipalities*					
Cumulative EVs	23.51	2	62.98	0	654
Charging stations	0.38	0	0.49	0	1
Population	4756.27	3549	3411.46	346	18,709
Household income	382,723.20	373,923.30	43,237.82	300,324.10	541,030.90
Detached houses	90.30	92.24	6.37	67.23	98.36
*Multi-station municipalities*					
Cumulative EVs	12.52	1	25.37	0	151
Charging stations	0.90	0	2.28	0	13
Population	4781.40	4060	3222.24	1003	11,723
Household income	374,043.80	373,378.30	28,878.90	303,889.10	461,981.80
Detached houses	87.03	90.04	10.14	58.06	97.25
*Donor municipalities*					
Cumulative EVs	10.51	1	35.84	0	395
Charging stations	0	0	0	0	0
Population	2879.55	2016	2879.07	196	18,850
Household income	370,738.70	367,781.60	36,625.30	285,091.80	841,848.80
Detached houses	92.04	93.85	5.56	68.83	100

Data sources are OFV (2018), NOBIL (2018), and SSB (2018). Mean municipal household income is measured in 2015 NOK, with 1 USD approx. 8 NOK in 2015. Detached houses is measured as the percentage of all households that are detached or duplex

Table 2 shows the difference in the outcome and treatment variables (EV numbers and charging stations available, respectively) between these 3 municipality groups across the entire observation period.^14^ Aside from differences in charging stations, cumulated EV registrations is higher in the two treatment groups than in the donor group. We further observe that the municipalities in these three groups are similar in terms of their population size, wealth, and urban density. In particular, while the mean donor population is lower than those of the treated groups, it is less than two-thirds of a standard deviation smaller. We observe that the support of observables for all three groups overlap.

### Panel Data Approach

The objective of our panel data strategy is to estimate the non-linear impacts of EV charging infrastructure on the number of EVs purchased. Our main outcome variable is the quantity of new cars registered, at the car model-level *m*, and across municipalities *i*, and quarters *t*. Our treatment variable is the number of charging stations (or alternatively charging points) available in a given municipality *i* and at a given time *t*.

Formally, our baseline panel data specification is given by:

1 $$\begin{aligned} \textit{ln(EV)}_{mit}= \alpha + \beta \, \textit{ln(chargers)}_{it} +\gamma \, X_{mit} + \delta _{mi} + \theta _t + \varepsilon _{mit}\,, \end{aligned}$$

where ${ln(EV)}_{mit}$ is the log of new cars registered by model, municipality and quarter, ${ln(chargers)}_{it}$ is the natural log of publicly accessible EV charging stations (or charging points).^15^$X_{mit}$ is a set of control variables including the log of a municipality’s mean household income and the gross list price of each car.^16^ We also further include two trend variables. First, we interact household income with a time-trend to allow for the income effect to change over time as the EV market becomes more mature. Second, we interact the quantity of hybrid vehicles registered in 2008 (before our sample period) with a time-trend to proxy for environmental preferences in each municipality. Next, we include municipality-model fixed effects $\delta _{mi}$, which capture model-specific preference heterogeneity across municipalities due to availability of certain brands, or practicality of certain car characteristics such as including battery range or different car styles. This further controls for municipality characteristics that are time-invariant and could affect EV demand, such as degree of urbanisation, toll road or bus lane prevalence, commuting behaviours, average driving distances, technological hesitancy, etc. Quarter fixed effects $\theta _t$ capture country-wide trends, including technological improvements in EV models (e.g. increased battery range) and changing competition environment across the country. Lastly, $\varepsilon _{mit}$ is a random error term.

One conceptual issue with equation (1) is the potential endogeneity of charging infrastructure. As discussed above, demand for EVs can be affected by various factors that vary across time and municipalities, and that also influence investments in chargers and therefore their quantity. Additionally, through reverse causality, a greater number of EVs in circulation could lead to more investments in EV charging stations.

In an attempt to address this problem, we exploit plausibly exogenous variation in the availability of public parking places in each municipality as part of an instrumental variable strategy. The first stage model is driven by the fact that public charging infrastructure generally requires space to park electric vehicles, so that available publicly regulated parking areas in a municipality increase the probability and level of treatment by providing locations for charger installations.

We further argue that the exclusion restriction, which requires that our instrument ${Z}_{it}$ affects EV purchases in any given municipality-quarter only through the variable ${ln(chargers)}_{it}$, is plausible. First, municipality fixed effects control for any time-invariant individual municipality effects. Second, we use the number of parking places in a fixed year, 2017, and specify a Bartik-type instrument (Bartik 1991) to generate exogenous temporal variation:

2 $$\begin{aligned} \textit{Z}_{it} = ln(\textit{car parks}_{i}) \times ln\left( \sum _{j, j\ne i}chargers_{j,t-1}\right) \,, \end{aligned}$$

where the first part of ${Z}_{it}$ is the log of publicly regulated parking places in municipality *i*, and the second is the lagged log of charging stations (or points) installed in all other municipalities. This yields the following first stage equation:

3 $$\begin{aligned} \textit{ln(chargers)}_{it}= \tau + \sigma Z_{it} + \pi X_{mit} + \psi _{mi} + \xi _t + \mu _{mit}\,, \end{aligned}$$

where the notation follows from above and $\mu _{mit}$ is a random error term.

This identification strategy is close to Li et al. (2017), who interact the log of the number of grocery stores with the lagged log of charging stations in other MSAs. Similarly, our instrument in Eq. 2 captures the exogenous national trend in charger installations, accounting for all national subsidies and incentives, as well as national-level shocks to costs, technologies, culture and policies, and interacts the municipal potential for installations. Intuitively, national-level trends affect municipalities differently based on their local characteristics, and municipalities with more abundant parking spaces are expected to be more likely to install charging infrastructure in response to national trends or shocks.

In order to document non-linearities presumably associated with network effects, we estimate a set of specifications using polynomial forms of the instrumented charger variable. For this purpose, we implement the CF approach discussed in Wooldridge (2015), whereby residuals from the first stage regression $\hat{\mu }_{mit}$ are included in the second stage to control for variability that is *not* associated with the instrumental variable:

4 $$\begin{aligned} \textit{ln(EV)}_{mit}= \alpha + f(chargers) + \gamma \, X_{mit} + \delta _{mi} +\theta _t + \rho \,\hat{\mu }_{mit} + \textit{e}_{mit}\,, \end{aligned}$$

where $f(\cdot )$ is a quadratic or cubic function.^17^

Finally, we also carry out the following robustness checks. First, we drop the car price from the estimation, so as to document concerns that endogeneity in this variable may affect our estimated elasticities.^18^ Second, we use the number of parking spaces in 2015 rather than 2017 to construct an alternative instrument and test it’s robustness to an alternative measure in the number of parking places.^19^ Third, we construct an alternative instrument that excludes neighboring municipalities, addressing potential concerns associated with regional effects. Fourth, we interact the treatment variable with a dummy for BEVs, and test for differences in the provision of charging infrastructure as compared to plug-in hybrids. Fifth, we add further control variables, namely municipal-level population, and level of urbanization. Sixth, we estimate a separate treatment elasticity for ‘early’ and ‘late’ periods of our sample, splitting between observations in 2010–2013 and 2014–2017.

Lastly, we separate treatment elasticity by the municipality’s degree of urbanisation—urban/city, suburban/town or rural. The fixed effects above capture time-invariant municipality differences such as access to toll roads and bus lanes, commuting methods, and overall EV preferences. However, some factors could lead to varied EV adoption responses to charger installation. For example, if acceptance of the new technology is lower in rural areas and evolves over time at a slower rate to in cities, this would not be captured by fixed effects. Furthermore, denser urban environments lend themselves to greater peer and network effects through proximity and ease of observation. Finally, the base of installed charging infrastructure varies considerably across degree of urbanisation. Thus if the above non-linear elasticity estimates are significant, this would directly follow through to mean treatment elasticity values in municipalities with greatly differing charger numbers.

### Synthetic Control Method

We now discuss the SCM approach, which allows us to estimate the impact of providing charging infrastructure in municipalities that previously had none. Specifically, we focus on 47 one-station municipalities that installed a single charging station, and on 17 multi-station municipalities that installed more than one station. For each treated unit, we construct a counterfactual “synthetic” unit by estimating a set of weights applied to the 110 municipalities with zero charging stations included in the donor pool. Intuitively, the weights are selected so as to minimize the distance between the pre-treatment outcome of the treated unit and that of the synthetic unit, and the latter is used as a counterfactual to quantify post-treatment differences with the treated unit.

Formally, in the SCM approach derived from Abadie and Gardeazabal (2003) and Abadie et al. (2010), for each treated municipality *j* (either in the one-station and multi-station groups) the outcome is the cumulative number of EV purchases EV$_{jt}$. We define a synthetic municipality as a weighted sum of the cumulative number of EV purchases EV$_{it}$ in all municipalities *i* of the donor pool:

5 $$\begin{aligned} \textit{EV}_{jt}^{{ SCM}} =\sum _{i} \omega _{ji}{} \textit{EV}_{it} \ , \end{aligned}$$

where $\omega _{ji}$ is the weight attributed for municipality *i* in constructing a synthetic control for treated municipality *j*. The weights result from minimization of the squared-sum of pre-treatment differences in our outcome variable, cumulative EV sales, between each synthetic and treated municipality—the mean squared prediction error (MSPE):

6 $$\begin{aligned}&\min _{\omega _{ji}} \ \ \sum _{t=0}^{T_0}\left( \textit{EV}_{jt} -\sum _{i} \omega _{ji} \textit{EV}_{it}\right) ^{2} \,\nonumber \\&\textit{s.t.} \ \sum _i \omega _{ji} = 1 \ , \quad \omega _{ji} \ge 0 \, , \end{aligned}$$

where $T_0$ is last quarter before treatment. Note that the quarter of treatment differs for each municipality, and thus the number of periods before and after treatment also varies (see “Appendix 1”). We therefore use a staggered design, where the analysis time-points are centred around each municipality’s period of treatment ($T_0+1$). We restrict our treated municipalities to those with at least 4 quarters pre-treatment and at least 4 post-treatment to allow for sufficient matching and comparison dimensions. The matching period is then the entire observed pre-treatment period available, ranging from 4 to 26 periods, with an average of 18.8. Having a relatively long matching period is desirable to minimise potential bias and the MSPE, while we simultaneously maintain maximum model sparsity through fitting only on the outcome variable (Abadie 2021).

Before treatment, the difference between observed cumulative EVs, $\hbox {EV}_{jt}$, and the counterfactual synthetic outcome $\hbox {EV}_{jt}^{{ SCM}}$ should be as close as possible to 0. Post-treatment, the difference between $\hbox {EV}_{jt}$ and $\hbox {EV}_{jt}^{{ SCM}}$, denoted $\phi _{t}$, measures the treatment effect. Formally we calculate:

7 $$\begin{aligned} \textit{EV}_{jt} = \phi _{t}D_{t} + \textit{EV}_{jt}^{{ SCM}} \ , \end{aligned}$$

where $D_{t}$ is the post-treatment period indicator. We repeat the above for every treated municipality in the 2 treatment groups, and show the variation in impacts between these, as well as the overall trend and average treatment effects.

Abadie and Imbens (2011) show, however, that the SCM is subject to a version of the curse of dimensionality, whereby the probability that the weights assigned achieve a perfect match between the synthetic and treated unit decreases with the dimension of the matching. This can lead to a bias in the estimated treatment effect. To overcome this the ridge-augmented SCM approach adds a bias-correction term derived from a ridge regression of post-treatment outcomes for donor units on pre-treatment outcome values. The estimated ridge regression coefficients, $\hat{\eta }$, are then introduced into the model as the bias correction (see Ben-Michael et al. 2018).^20^ Formally, the ridge-augmented SCM weights are derived from:

8 $$\begin{aligned} \textit{EV}_{jt}^{{ RASCM}} = \sum _{i} \omega _{ji}^{{ RASCM}} \, \textit{EV}_{it} +\left( \textit{Y}_{j} -\sum _{i} \omega _{ji}^{{ RASCM}} \textit{Y}_{i}\right) \cdot \hat{\eta } \end{aligned}$$

where *Y* is the vector of pre-treatment cumulative EVs, and $(\textit{Y}_{j} - \sum _{i} \omega _{ji} \textit{Y}_{i})$ is an estimate of the SCM bias. Importantly, the ridge-augmented SCM weights $\omega _{ji}^{RASCM}$ are not constrained to be positive, which provides additional flexibility for fitting pre-treatment outcomes. Ben-Michael et al. (2018) show that the ridge-augmented SCM achieves smaller pre-treatment residuals, and in turn generates a more accurate estimate of the treatment effect. In our results, we focus primarily on the ridge-augmented SCM results and report the standard SCM results in “Appendix 3” for comparison.

We further conduct extensive robustness analysis of our ridge-augmented SCM results. Consistent with the SCM literature, these take the form of placebo tests where certain aspects of treatment assignment are changed in order to rule out spurious effects (Abadie and Gardeazabal 2003). First, we carry out a spatial placebo analysis, where observed treatment interventions are iteratively reassigned to every untreated municipality in the donor pool, generating placebo treatment corresponding to the treatment dates among treated municipalities. From this we are able to compute p-values for our original estimates (see also Abadie et al. 2015; Andersson 2019). Specifically, the p-value is calculated as the proportion of placebo estimates that are at least as large as the average treatment effect estimated for treated municipalities.^21^

As a second robustness check, we conduct a set of temporal placebo tests (Abadie et al. 2015), based on Heckman and Hotz (1989) and Bertrand et al. (2004). Specifically, for each treated municipality we shift the treatment period a year (4 quarters) earlier and estimate the ridge-augmented SCM weights. In other words, the pre-treatment matching period is reduced by four quarters in order to check that the estimated effect is not spurious. If we observe systematic, sizeable differences between treated and synthetic outcomes after the artificial treatment period, this would provide evidence against the ridge-augmented SCM estimates.

Finally, we conduct a version of the “leave-one-out” test (Abadie et al. 2015; Andersson 2019) to assess the potential influence of urban-proximate municipalities in the donor pool. Specifically, we remove any donor group municipalities that have a weekday morning driving-time proximity to cities and urban municipalities, based on the SSB (2018) classification, and repeat the ridge-augmented SCM analysis to remove potential bias from EV adoption incentives in these untreated units due to commuting ties, toll road use, or similar. If we observe large differences in the estimated treatment effects, we would potentially be concerned about bias in our primary results by not explicitly accounting for such non-charger incentives. Such a bias, though, would theoretically reduce our treatment effect estimations, meaning we originally underestimate or find a lower-bound estimate.

## Estimation Results

This section reports our empirical results. First, we present the panel data analysis, documenting non-linear impacts of EV charging infrastructure on the number of EVs purchased. Second, we discuss results from the ridge-augmented SCM, and document the impact of initial charging infrastructure provision on cumulative EV sales.

### Panel Data Results

We start by estimating a set of linear specifications (Eq. 1), which closely align with the work of Li et al. (2017). Next, we consider non-linear specifications based on polynomial function of charging stations (Eq. 4). Lastly, we report robustness results.

#### Linear Specifications

Our estimation results from the linear models are reported in Table 3. In columns (1), we report OLS estimates for a regression of the log of EV registrations on the log of charging stations. In column (2) we report results for the same function estimated with 2-stage least squares (2SLS). Columns (3) and (4) repeat this sequence, with charging points as the treatment variable instead of charging stations. All models include quarter and municipality-model fixed effects, and standard errors are clustered at the municipality level and reported in parentheses. First-stage results for the 2SLS specifications are provided in “Appendix 2”, Table 12.

**Table 3:** Baseline results from panel data estimation

	Charging stations		Charging points	
	OLS	2SLS	OLS	2SLS
	(1)	(2)	(3)	(4)
ln(charging stations)	− 0.008	0.126**	–	–
	(0.006)	(0.054)		
ln(charging points)	–	–	− 0.004	0.074***
			(0.003)	(0.026)
ln(car price)	0.108***	0.110***	0.108***	0.110***
	(0.008)	(0.008)	(0.008)	(0.008)
ln(income)	− 0.007	− 0.036	− 0.007	− 0.025
	(0.092)	(0.109)	(0.092)	(0.110)
ln(income) × time	− 0.0002	0.0001	− 0.0003	− 0.0001
	(0.005)	(0.005)	(0.005)	(0.005)
ln(hybrids) × time	0.008***	0.008***	0.008***	0.008***
	(0.001)	(0.001)	(0.001)	(0.001)
Constant	− 1.298	− 1.370	− 1.290	− 1.348
	(1.090)	(1.314)	(1.089)	(1.334)
N	367,984	366,296	367,984	366,296
Within-R$^2$	0.0779	0.0675	0.0779	0.0646
1st-stage partial F-stat.	–	19.01	–	25.54

In all columns, the dependent variable is the log of new electric vehicle registrations (${ln(EV)}_{mit}$). Columns (1) and (2) consider charging stations as the treatment variable, and columns (3) and (4) instead use charging points. All specifications include quarter and municipality-model fixed effects. The 1st stage partial F-statistic for the instrumental variable (columns (2) and (4)) are derived from first-stage regression reported in “Appendix 2”, Table 12. Standard errors clustered at the municipality level reported in parentheses

*, ** and *** respectively denote significance at 10%, 5% and 1% levels

OLS results in column (1) indicate no statistically significant effect of charging stations on EV purchases. Comparing this to the 2SLS specification in column (2), suggests a negative endogeneity bias. Our IV specification in column (2) shows a highly significant estimated elasticity of charging stations on EVs of 0.126. Furthermore, our instrument interacting parking spaces with trends in national charger availability has significant explanatory power over the quantity of charging stations available in a given municipality-quarter, with a first-stage F-statistic associated with the instrument of 19.01. A comparison of columns (3) and (4) confirms a downward bias associated with OLS estimation, with the 2SLS estimate for the elasticity of charging points on EVs of 0.074. The F-statistic associated with the instrument for charging points in the first-stage regression is 25.54.

Our results show that the elasticity with respect to charging points is almost half the magnitude of the elasticity for charging stations. This suggests that consumers respond more on average to the simple visual presence of stations than to the specific number of plugs available. That is, ceteris paribus, constructing more EV charging stations with fewer points each would tend to engender more EV purchases than installing fewer stations with more points each. This is consistent with a psychological reassurance effect that the charging station network provides to curbing drivers’ range anxiety.

#### Non-linear Specifications

Table 4 reports results from the polynomial forms using a CF approach (Eq. 4). Columns (1) to (3) respectively provide linear, quadratic, and cubic model estimates with charging stations as the treatment variable. Columns (4) to (6) repeat the same sequence of estimations but using charging points as the treatment variable. In all models we additionally include quarter and municipality-model fixed effects. Standard errors are clustered at the municipality level, bootstrapped with 500 replications, and reported in parentheses.^22^

**Table 4:** Results from control function estimation

	Charging stations			Charging points		
	Linear	Quadratic	Cubic	Linear	Quadratic	Cubic
	(1)	(2)	(3)	(4)	(5)	(6)
ln(charging stations)	0.126***	0.043	0.131***	–	–	–
	(0.045)	(0.041)	(0.046)			
ln(charging stations)$^2$	–	0.046***	− 0.036	–	–	–
		(0.008)	(0.029)			
ln(charging stations)$^3$	–	–	0.018**	–	–	–
			(0.009)			
ln(charging points)	–	–	–	0.074***	0.025	0.132***
				(0.021)	(0.020)	(0.021)
ln(charging points)$^2$	–	–	–	–	0.015***	− 0.055***
					(0.003)	(0.094)
ln(charging points)$^3$	–	–	–	–	–	0.012***
						(0.002)
ln(car price)	0.110***	0.110***	0.110***	0.110***	0.110***	0.110***
	(0.008)	(0.008)	(0.008)	(0.008)	(0.008)	(0.008)
ln(income)	− 0.036	− 0.099	− 0.130	− 0.025	− 0.057	− 0.108
	(0.100)	(0.087)	(0.080)	(0.103)	(0.091)	(0.077)
ln(income) × time	0.0006	0.002	0.003	− 0.0001	0.001	0.002
	(0.005)	(0.005)	(0.004)	(0.005)	(0.005)	(0.004)
ln(hybrids) × time	0.008***	0.006***	0.006***	0.008***	0.007***	0.006***
	(0.001)	(0.001)	(0.001)	(0.001)	(0.001)	(0.0005)
First stage residuals	− 0.137***	− 0.127***	− 0.136***	− 0.080***	− 0.071***	− 0.079***
	(0.046)	(0.039)	(0.038)	(0.021)	(0.019)	(0.018)
Constant	− 1.370	− 1.288	− 1.062	− 1.348	− 1.389	− 1.118
	(1.214)	(1.256)	(1.127)	(1.211)	(1.201)	(1.033)
N	366,296	366,296	366,296	366,296	366,296	366,296
Adjusted within-R$^2$	0.0778	0.0796	0.0817	0.0779	0.0790	0.0826
1st-stage partial F-stat.	19.01	19.01	19.01	25.54	25.54	25.54

In all columns, the dependent variable is the log of new electric vehicle registrations (${ln(EV)}_{mit}$). Columns (1)–(3) consider charging stations as the treatment variable, and columns (4)–(6) instead use charging points. All specifications include quarter and municipality-model fixed effects. The 1st stage partial F-statistic for the instrumental variable is derived from first-stage regression results reported in “Appendix 2”, Table 12. Standard errors bootstrapped with 500 replications and clustered at the municipality level reported in parentheses

*, ** and *** respectively denote significance at 10%, 5% and 1% levels

**Fig. 2:**
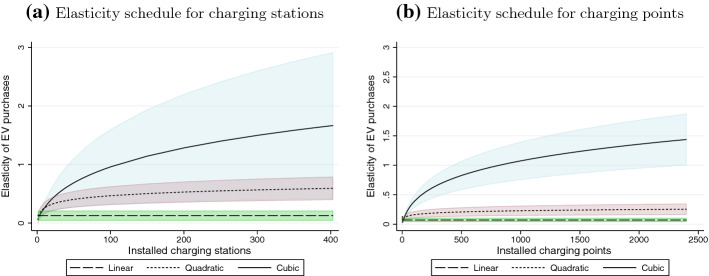
Elasticity of electric vehicle registrations as a function of the charging infrastructure. *Notes:* Based on the model estimates shown in Table 4. The graphed lines provide point elasticity estimates, and the shaded areas cover the 95% confidence intervals

Based on the overall model fit, our preferred model for charging stations is the cubic form (column 3), and we illustrate the implied schedule for elasticity estimates in Fig. 2a (panel a). At low values for the installed stock of charging stations, the elasticity of chargers on EV purchases is similar across specifications (e.g. at the sample mean of 3.09 charging stations the cubic specification gives an elasticity of 0.22). However, cubic polynomial results indicate a significant increase in the elasticity of charging stations on EV purchases as the stock of installed stations rises. At around 100 charging stations available, the elasticity is approximately unity, although the rise in elasticity for each additional installed station quickly diminishes.

Interestingly, our non-linear results also provide a rejoinder with the elasticity estimates of about 0.8 reported in Li et al. (2017), which refer to 353 MSAs with relatively significant EV sales over the period from 2011 to 2013. These MSAs also feature a stock of installed chargers of 22.13, which is significantly larger than what we have in our sample. Evaluating the polynomial function for a stock of installed chargers of 22, we obtain an elasticity of 0.54.

Results for charging points (Table 4 columns (4) to (6) and Fig. 2b also support an increasing elasticity schedule as the number of available charging points rises, although at a declining rate. In our preferred cubic specification (column 6), the elasticity evaluated at the mean value of charging point availability (13.46) is 0.12. At 200 charging points, the elasticity is around 0.57, and surpasses unity for a stock of around 800. Overall, the consumer reaction to a marginal increase in charging points is smaller compared to an increase in charging stations, which further supports the behavioral bias discussed previously.

**Fig. 3:**
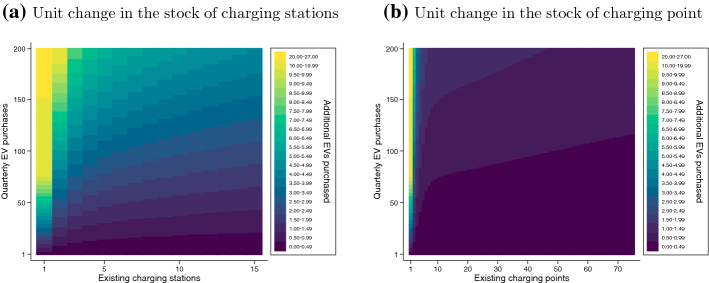
Electric vehicle registrations associated with incremental charging infrastructure. *Notes:* Based on the cubic model estimates shown in Table 4. This shows the number of new EVs registered after the installation of a single charging station (**a**) or point (**b**), across varying levels of existing infrastructure and previous EV purchases. “Quarterly EV purchases” refers to the quantity in the period before the charger installation

Implications of cubic specifications are further illustrated in Fig. 3, which reports the impact of a 1-station increment (panel a) and a 1-point increment (panel b) on EV registrations across varying levels of existing infrastructure and EV purchasing. This shows that the largest impact from installing an additional charging station is at a low level of existing infrastructure, and that the impact increases with the number of EVs purchased in the quarter just before installation. As the existing stock of stations grows, the additional EVs generated by further incremental installations diminishes. The pattern for charging points is similar, although the consumer reaction declines more rapidly than for charging stations, which is in line with a behavioral difference between charging stations and points discussed above.

#### Robustness Checks for Panel Data Estimation

Next, we report robustness checks for charging stations (Table 5) and points (Table 6). In both tables, column (1) reports results excluding the car price variable; column (2) uses 2015 parking spaces to construct the instrument instead of 2017; in column (3) the instrument excludes each municipality’s neighbors; column (4) adds the interaction between chargers and BEVs; column (5) adds extra control variables; column (6) allows the treatment elasticity to vary between early and late periods in our dataset; and column (7) estimates different treatment elasticities for urban, suburban or rural areas. For simplicity and ease of interpretation we focus on linear specifications, and provide estimates of our preferred cubic specifications in appendix Table 13 and Table 14.^23^ All models are estimated with a CF procedure and bootstrapped standard errors (500 replications) clustered at the municipality level are reported in parentheses. First stage results for all specifications are reported in Tables 15 and 16 of “Appendix 2” for charging stations and points, respectively.

**Table 5:** Alternative panel data specifications—charging stations

	No price	2015 parking	No neighbours	Chargers x BEV	Additional controls	Chargers × time	Chargers × urban
	(1)	(2)	(3)	(4)	(5)	(6)	(7)
ln(charging stations)	0.126***	0.139**	0.140***	0.121***	0.131***	–	–
	(0.046)	(0.055)	(0.048)	(0.046)	(0.045)		
ln(charging stations) × BEV	–	–	–	0.011**	–	–	–
				(0.005)			
ln(charging stations) × early	–	–	–	–	–	0.130***	–
						(0.046)	
ln(charging stations) × late	–	–	–	–	–	0.128***	–
						(0.045)	
ln(charging stations) × urban	–	–	–	–	–	–	0.289**
							(0.125)
ln(charging stations) × town	–	–	–	–	–	–	0.146***
							(0.047)
ln(charging stations) × rural	–	–	–	–	–	–	0.124***
							(0.042)
ln(car price)	–	0.110***	0.110***	0.108***	0.110***	0.110***	0.110***
		(0.008)	(0.008)	(0.007)	(0.008)	(0.008)	(0.009)
ln(income)	− 0.036	− 0.038	− 0.039	− 0.036	− 0.065	− 0.035	− 0.003
	(0.096)	(0.095)	(0.089)	(0.095)	(0.098)	(0.095)	(0.099)
ln(income) × time	0.001	0.001	0.001	0.001	0.002	0.001	− 0.001
	(0.005)	(0.004)	(0.004)	(0.004)	(0.005)	(0.005)	(0.005)
ln(hybrids) × time	0.008***	0.008***	0.008***	0.008***	0.008***	0.008***	0.007***
	(0.001)	(0.001)	(0.001)	(0.001)	(0.001)	(0.001)	(0.001)
ln(population)	–	–	–	–	− 0.034	–	–
					(0.122)		
Proportion of detached and duplex dwellings	–	–	–	–	0.003	–	–
					(0.003)		
First stage residual	− 0.137***	− 0.149***	− 0.150***	− 0.136***	− 0.142***	− 0.139***	− 0.140***
	(0.046)	(0.055)	(0.048)	(0.046)	(0.045)	(0.045)	(0.042)
Constant	0.051	− 1.382	− 1.383	− 1.346	− 1.368	− 1.139	− 1.108
	(1.190)	(1.163)	(1.169)	(1.232)	(1.631)	(1.269)	(1.218)
N	366,296	366,296	366,296	366,296	366,296	366,296	366,296
Adjusted within-R$^2$	0.0767	0.0778	0.0779	0.0779	0.0779	0.0778	0.0778
1st-stage partial F-stat.	18.32	11.29	16.80	19.01	19.51	19.01	19.01

In all columns, the dependent variable is the log of new electric vehicle registrations (${ln(EV)}_{mit}$). Column (1) omits the car price variable. Column (2) uses the number of parking spaces in 2015 to construct the instrument. Column (3) excludes neighboring municipalities to construct the instrument. In column (4), we interact the treatment variable with a dummy for battery-only EVs. Column (5) includes further control variables. In column (6), we estimate separate elasticities for observations in 2010–2013 and 2014–2017. Column (7) estimates separate elasticities by municipal degree of urbanisation—urban/city, town/suburban, rural. All specifications are estimated with a control function approach and include quarter and municipality-model fixed effects. The 1st stage partial F-statistic for the instrumental variable is derived from first-stage regression results reported in “Appendix 2”, Table 15. Standard errors bootstrapped with 500 replications and clustered at the municipality level reported in parentheses

*, **, *** respectively denote significance at 10%, 5% and 1% levels

**Table 6:** Alternative panel data specifications—charging points

	No price	2015 parking	No neighbours	Chargers x BEV	Additional controls	Chargers × time	Chargers × urban
	(1)	(2)	(3)	(4)	(5)	(6)	(7)
ln(charging points)	0.074***	0.087***	0.079***	0.072***	0.077***	–	–
	(0.020)	(0.026)	(0.022)	(0.021)	(0.022)		
ln(charging points) × BEV	–	–	–	0.005*	–	–	–
				(0.003)			
ln(charging points) × early	–	–	–	–	–	0.081***	–
						(0.023)	
ln(charging points) × late	–	–	–	–	–	0.076***	–
						(0.022)	
ln(charging points) × urban	–	–	–	–	–	–	0.134
							(0.146)
ln(charging points) × town	–	–	–	–	–	–	0.083***
							(0.026)
ln(charging points) × rural	–	–	–	–	–	–	0.073***
							(0.022)
ln(car price)	–	0.110***	0.110***	0.108***	0.110***	0.110***	0.110***
		(0.008)	(0.008)	(0.008)	(0.008)	(0.008)	(0.006)
ln(income)	− 0.025	− 0.029	− 0.027	− 0.025	− 0.059	− 0.025	− 0.019
	(0.097)	(0.100)	(0.099)	(0.097)	(0.103)	(0.098)	(0.098)
ln(income) × time	− 0.0001	− 0.0002	− 0.0002	− 0.0001	0.001	− 0.0003	− 0.000
	(0.005)	(0.005)	(0.005)	(0.005)	(0.005)	(0.005)	(0.005)
ln(hybrids) × time	0.008***	0.008***	0.008***	0.008***	0.008***	0.008***	0.008***
	(0.001)	(0.001)	(0.001)	(0.001)	(0.001)	(0.001)	(0.001)
ln(population)	–	–	–	–	− 0.049	–	–
					(0.124)		
Proportion of detached and duplex dwellings	–	–	–	–	0.003	–	–
					(0.002)		
First stage residual	− 0.080***	− 0.093***	− 0.085***	− 0.080***	− 0.083***	− 0.083***	− 0.080***
	(0.020)	(0.026)	(0.022)	(0.021)	(0.022)	(0.022)	(0.021)
Constant	0.074	− 1.364	− 1.355	− 1.326	− 1.259	− 1.427	− 1.329
	(1.234)	(1.170)	(1.186)	(1.166)	(1.577)	(1.220)	(1.180)
N	366,296	366,296	366,296	366,296	366,296	366,296	366,296
Adjusted within-R$^2$	0.0768	0.0779	0.0780	0.0780	0.0780	0.0780	0.0780
1st-stage partial F-stat.	24.57	14.17	23.73	25.54	23.04	25.54	25.54

In all columns, the dependent variable is the log of new electric vehicle registrations (${ln(EV)}_{mit}$). Column (1) omits the car price variable. Column (2) uses the number of parking spaces in 2015 to construct the instrument. Column (3) excludes neighboring municipalities to construct the instrument. In column (4), we interact the treatment variable with a dummy for battery-only EVs. Column (5) includes further control variables. In column (6), we estimate separate elasticities for observations in 2010–2013 and 2014–2017. Column (7) estimates separate elasticities by municipal degree of urbanisation—urban/city, town/suburban, rural. All specifications are estimated with a control function approach and include quarter and municipality-model fixed effects. The 1st stage partial F-statistic for the instrumental variable is derived from first-stage regression results reported in “Appendix 2”, Table 16. Standard errors bootstrapped with 500 replications and clustered at the municipality level reported in parentheses

*, **, *** respectively denote significance at 10%, 5% and 1% levels

Starting with results for charging stations (Table 5), we find that the elasticity estimates remain close to our primary linear elasticity estimate of 0.126, and the partial F-statistics associated with the instrument are also very similar across specifications. This suggests that endogeneity in the car price variable does not influence our elasticity of interest (column 1). Using parking space data for 2015 (column 2) or removing neighboring municipalities from the instrument (column 3) have only minor effects on the elasticity estimates, which reinforces our confidence in the instrument. Similarly, changing the set of controls (column 5) also has very little impact on the elasticity estimates, and population and urbanization are not statistically significant at conventional levels. This suggests that our control strategy, which closely follows Li et al. (2017), already captures these potential drivers of EV purchases. Interacting the treatment variable with an indicator for BEVs (column 4) suggests that the elasticity for BEVs is slightly larger (p-value <0.05). Column (6) suggests no significant difference in the treatment effect for early and late time periods.

We finally find the treatment effect does vary significantly with urbanisation. Rural areas, accounting for three quarters of observations, have a similar elasticity estimate to the main specification, with intermediate regions (towns or suburbs) slightly more elastic. Cities and urban areas, under 3% of observations, display a significantly greater reaction to new charging stations, with a more than doubled treatment elasticity of 0.289. This is directly related to the level of charging infrastructure available in each municipality group, as demonstrated in our previous non-polynomial model. As discussed above, our charger elasticities increase the larger the base of installed stations. Urban municipalities have a mean of 41 charging stations in our sample, compared to 5 and 1 in intermediate and rural municipalities, respectively. This means that, as per our polynomial findings, the urban-rural elasticities will also vary along with their respective stages of charging infrastructure build-up. The varied responses to charger installations could further be indicative of behaviors and preferences that are not captured in the municipality fixed effects, such as an overall hesitancy towards the technology that dampens reactions to incentives such as charger installations, a lesser importance of public chargers due to more prevalent home charging ability, or a relatively greater impact of visibility, and peer and network effects in denser, urban environments.

Results for charging points (Table 6) follow the same logic, and elasticity estimates from alternative specifications do not part significantly from the primary linear model’s 0.074. Column (1) suggests that results do not suffer from otherwise unaccounted endogeneity through the vehicle price, and columns (2) and (3) show that our instrument stands up to changes in both halves of the Bartik construction. We also observe insignificant changes when we add an interaction term for BEVs (column 4), control variables (column 5), and check for differences between early-period and late-period elasticities (column 6). Rural and sub-urban areas follow the same pattern as for stations, given greater mean charging point numbers the more urban the municipality classification (respectively 209, 20 and 4 points in urban, sub-urban and rural areas). However the treatment elasticity for charging points in urban areas, while also larger, cannot be precisely estimated (column 7). Overall, each of these alternative specifications supports our primary estimations and the strength of our instrument.

### Synthetic Control Results

We now report results from the SCM approach, quantifying how cumulative EV purchases respond to the installations of the first charging station(s). We focus on results from the ridge-augmented SCM, which tends to generate smaller pre-treatment residuals, and report results for the traditional SCM approach in “Appendix 3”. We then follow with a set of placebo tests to document robustness of the analysis.

#### Pre-treatment Matching and Treatment Effect Estimates

**Fig. 4:**
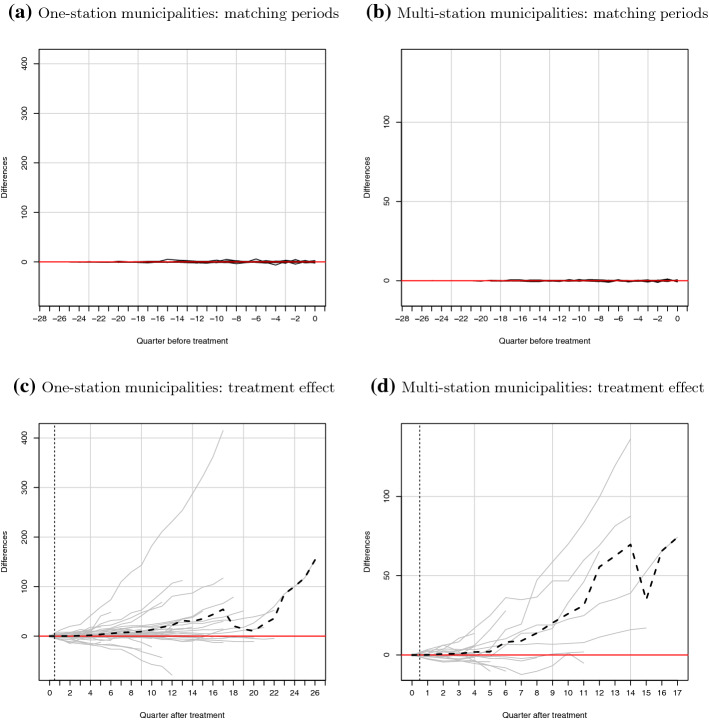
Gap in cumulative EV stock between treated municipalities and synthetic controls. *Notes:* The solid gray lines represent the ridge-augmented SCM estimated differences between each treated municipality and its synthetic counterpart. The black dashed lines present the mean differences across treated units

Figure 4a, b, present our ridge-augmented SCM estimation results for pre-treatment matching periods for the one-station treatment group and multi-station treatment group, respectively. These show that, for both treatment groups, the differences between the numbers of EVs registered in each treated and its synthetic municipality prior to the charger installation shock is close to zero across the pre-treatment periods. This suggests that synthetic control units precisely track EV registrations in the pre-treatment period, and provides confidence that synthetic municipalities provide credible counter-factual information for each treated municipality in the absence of charging infrastructure.^24^

**Table 7:** Summary of post-treatment synthetic control results

Quarter post-treatment	One-station municipalities			Multi-station municipalities		
	Obs.	Mean	Median	Obs.	Mean	Median
1	47	0.20	$-$0.08	17	0.01	$-$0.05
2	47	0.18	0.04	17	0.64	$-$0.14
3	47	0.74	0.50	17	0.82	0.59
4	47	1.68	0.52	17	1.85	$-$0.58
5	46	3.50	0.66	13	2.07	$-$0.93
6	38	5.31	1.88	10	8.12	6.45
7	36	7.19	3.83	8	8.86	10.06
8	35	7.96	3.65	8	13.81	10.53
9	33	9.15	4.97	7	20.22	16.71
10	30	12.81	5.99	7	25.99	22.60
11	26	17.85	6.63	7	31.23	24.80
12	24	22.08	9.95	5	55.53	65.42
13	20	31.31	12.09	4	62.40	58.28
14	18	29.67	8.80	4	69.68	63.26
15	17	34.91	4.98	2	34.80	34.80
16	14	43.66	9.40	1	65.60	65.60
17	13	54.09	14.25	1	74.20	74.20
18	8	20.64	12.37			
19	7	14.25	11.76			
20	6	10.20	5.88			
21	4	24.31	29.82			
22	3	35.81	52.23			
23	1	85.06	85.06			
24	1	100.16	100.16			
25	1	117.87	117.87			
26	1	153.76	153.76			

This table summarizes results for the post-treatment gap in cumulative EV stock between treated municipalities and synthetic controls. Mean and median reported refer to the distribution of treatment effects estimated from the ridge-augmented SCM

In Fig. 4c, d, we report post-treatment quarterly differences between numbers of EVs in a treated municipality and the corresponding number for the estimated synthetic municipality, for the one-station and multi-station treatment groups, respectively. We also plot the average treatment effect across treated municipalities as a dashed line, and provide the mean and median differences between treated and synthetic municipalities for each post-treatment quarter in Table 7.^25^

Overall, results suggest that the provision of an initial charging station has a positive impact on EV registrations. Quantitatively, we estimate a one-station average treatment effect of 1.7 extra EVs registered four quarters post-installation. Eight quarters post-treatment, the estimated difference rises to 8.0 more EVs than would otherwise be registered. This is equal to 5.4 and 21.7% more EVs than would otherwise have been bought after one and two years, respectively. Evidence further suggests that the impact increases with the size of the shock—the multi-station average treatment effect is larger. Four quarters after the first installation, this group had on average 1.9 additional EVs registered. We also observe an upward trend in the treatment effect, as the average difference between treated and synthetic units increases to 13.8 extra EVs 2 years post-treatment. The positive treatment effect associated with multi-station installations amounts to about 8.0 and 46.1% more EVs on average, respectively.

These results also match the panel data findings above in Sect. 3.1.2, where we see the immediate impact of the first charging infrastructure is low when there are few EVs previously registered. Applying the single-increment results from Sect. 3.1.2 and Fig. 3 to the two SCM municipality groups, we find that the installation of the first charging station in the one-station group generated approximately 0.39 new EVs, on average, directly in the period of installation. For the multi-station group, an average of 3 charging stations were installed in the initial phase, and we therefore find that this lead to an average of around 0.78 additional EVs being purchased in the initial treatment period. Moreover, we find that when there is no existing charging infrastructure, the installation of the first charging point has a similar impact to that of the first station. Fundamentally, we see it takes time for the network dynamics to play out and the full benefits of early charger provision to be seen.

#### Robustness: Placebo Tests for Synthetic Control Results

**Fig. 5:**
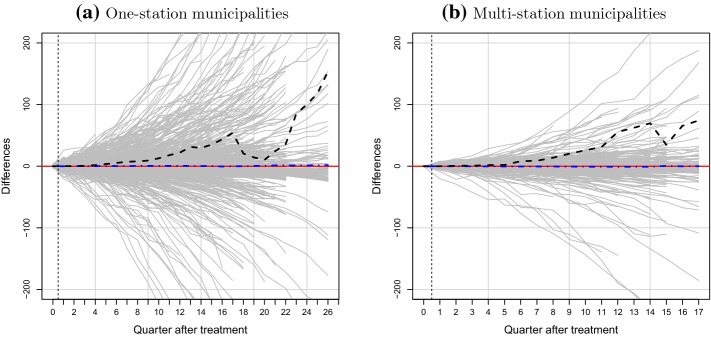
Synthetic control results for the spatial placebo tests. *Notes:* This figure shows the results for the spatial placebo tests, comparing average gap in cumulative EV stock between treated municipalities and synthetic controls with placebo gaps for the control municipalities. The solid grey lines represent the placebo difference estimates for donor pool municipalities. The black dashed lines provide the mean difference estimates for the treated municipalities from Fig. 4. The dashed-dotted lines give the means of the placebo estimates. See Appendix C, Table C2, for the underlying data

We now we present the results of placebo tests to document robustness of our SCM findings. As described above, we first conduct a set of spatial placebo tests, with results shown in Fig. 5 for one-station municipalities (Fig. 5a) and multi-station municipalities (Fig. 5b). In both panels, individual placebo estimates of EV number differences are displayed in gray, while the dashed-dotted line shows the average placebo ‘treatment effect’ for comparison to the black dashed line with the average treatment effect of our treated municipalities.^26^

The estimated placebo differences in EV purchases exhibit significant heterogeneity, although the average placebo treatment effect for both one-station and multi-station is estimated to be consistently close to 0. This is also shown in Table 8, which provides per period average and median spatial placebo estimates for each group. We also report an estimate of the p-value associated with the average treatment effect reported in Table 7, each period, as measured by the share of placebo estimates that are larger than the average treatment effect estimated on treated municipalities.

**Table 8:** Summary results for spatial placebo tests

Quarter post-treatment	One-station municipalities				Multi-station municipalities			
	Obs.	Mean	Median	p-value	Obs.	Mean	Median	p-value
1	2193	0.02	− 0.08	0.244	856	− 0.06	− 0.11	0.293
2	2193	0.04	− 0.15	0.311	856	− 0.11	− 0.30	0.263
3	2193	0.08	− 0.23	0.255	856	− 0.18	− 0.40	0.251
4	2193	0.14	− 0.28	0.204	856	− 0.27	− 0.43	0.189
5	2086	0.23	− 0.34	0.150	773	− 0.38	− 0.34	0.189
6	1977	0.29	− 0.47	0.123	688	− 0.48	− 0.48	0.063
7	1867	0.38	− 0.66	0.111	598	− 0.44	− 0.74	0.074
8	1757	0.47	− 0.65	0.118	598	− 0.51	− 0.87	0.050
9	1648	0.51	− 0.94	0.126	509	− 0.74	− 1.17	0.037
10	1539	0.51	− 1.21	0.110	509	− 0.75	− 1.54	0.033
11	1430	0.54	− 1.81	0.092	509	− 0.83	− 1.64	0.033
12	1320	0.47	− 2.16	0.084	411	− 0.99	− 2.03	0.024
13	1210	0.49	− 2.83	0.069	312	− 0.95	− 1.92	0.029
14	1100	0.40	− 2.86	0.080	312	− 0.98	− 2.27	0.029
15	990	0.23	− 3.59	0.085	211	0.59	− 3.07	0.076
16	880	− 0.44	− 4.82	0.076	107	− 0.17	− 5.15	0.056
17	770	− 0.03	− 5.69	0.075	107	− 0.04	− 5.57	0.056
18	660	− 0.05	− 6.57	0.155				
19	550	0.49	− 7.40	0.187				
20	440	0.71	− 8.13	0.209				
21	330	1.29	− 8.98	0.148				
22	220	1.34	− 9.36	0.118				
23	110	1.15	− 9.38	0.082				
24	110	1.24	− 10.91	0.055				
25	110	1.37	− 10.65	0.064				
26	110	1.62	− 12.02	0.055				

This table summarizes results for the post-treatment gap in cumulative EV stock for non-treated municipalities subject to placebo treatments corresponding to the treatment dates among treated municipalities, and synthetic controls. We report mean and median placebo treatment effects. The p-values represent the proportion of placebo difference estimates that are at least as large as the average treatment effect for treated municipalities

Results generally indicate that the significance of our average treatment effect increases over time. For one-station municipalities, the treatment effect estimates for treated municipalities are marginally significant, and we find that it is below a 10% threshold between the 11th and the 18th quarters.^27^ Results for multi-station municipalities provide further evidence for the greater impact of a larger treatment, as the p-value for the multi-station average treatment effect falls under 0.10 in the 6th quarter post-treatment, and below the 0.05 threshold after 2 years.

**Fig. 6:**
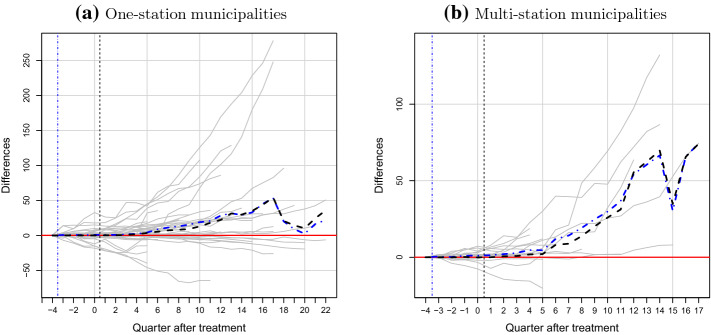
Synthetic control results for the temporal placebo tests. *Notes:* This figure shows the results for the temporal placebo tests, comparing average gap in cumulative EV stock between treated municipalities and synthetic controls with those derived with an artificial 4-quarter earlier treatment. The solid grey lines represent the estimated differences between each treated municipality and its synthetic counterpart, with a placebo installation of charging stations 4 quarters prior to the actual installation. The black dashed lines provide the mean difference estimates for the treated municipalities from Fig. 4. The dashed-dotted lines present the means of the placebo estimates. See “Appendix 3”, Table 18, for the underlying data

Results for the second placebo test are reported in Fig. 6, which shows our temporal placebo results. The solid grey lines present the individual placebo estimates generated by giving each municipality an artificial treatment 4 quarters prior to the observed one. The dashed-dotted line shows the mean placebo differences, and the black dashed line the original SCM average treatment effect estimates. Table 18 in the appendix provides the means and medians of the temporal placebo estimates for the two treatment groups.

Results suggest that the mean placebo differences remain close 0 in the initial 4 placebo treatment periods. After the true treatment period, the temporal placebo average closely follows our average treatment effect estimates. The small differences from the original estimates can be explained by the use of a shorter matching period, which implies slightly different weights attributed to donor municipalities. This provides further confidence that our estimated difference in EV purchases can be attributed to the early installation of charging infrastructure at observed dates.

**Fig. 7:**
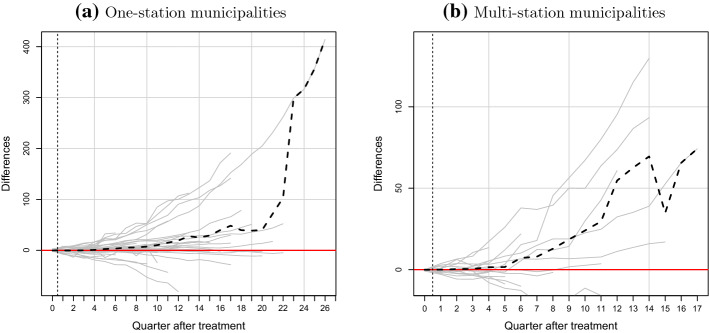
Synthetic control results for “leave-one-out” tests. *Notes:* This figure shows the results for the “leave-one-out” test. This is a repeat of Fig. 4c, d, excluding six city-proximate donor group municipalities 19. The solid gray lines represent the ridge-augmented SCM estimated differences between each treated municipality and its synthetic counterpart. The black dashed lines present the mean differences across treated units

The results of the final robustness check, the “leave-one-out” tests that omit certain urban-proximate municipalities, are shown in Fig. 7. This provides little evidence that our main results are sensitive to the presence of urban-proximate municipalities in the donor pool. We find six donor group municipalities that are less than a one hour drive to a city or urban municipality (see Table 19).^28^ The exclusion of these units from the donor pool does not greatly impact the estimations except for the one-station estimated differences between periods 18 and 22, where the treatment estimate is larger, and then from period 23, where only one treated municipality remains, with a much larger estimated difference. Specifically, at the mean, we estimate here 0.9 and 5.7 more EVs purchased by periods quarters 4 and 8, respectively, in the one-station group. This is slightly lower than the original estimates, though the medians remain almost identical. For the multi-station treatment group, we estimate an effect of 1.5 and 12.9 EVs by quarters 4 and 8, respectively. This is very close to the original estimates. Again, the medians remain similar. We provide the full “leave-one-out” estimates in Table 20.

## Discussion and Conclusions

In this study, we have provided novel empirical evidence on the impact of EV charging infrastructure on the adoption of EVs, focusing on how the size of the infrastructure network affects the response of consumers. Our work is based on fine-scale temporal and geographical data for Norway, from the emergence of the market and the early movers of 2010 to the mature market with large market share by 2017.

Our results provide a first account of consumer response to infrastructure in locations that previously had none. We show that the very first charging station installations initially induce a small response by consumers, although a one-off shock has a lasting, increasing impact over time after installation. We have also shown that the size of the initial installation shock matters, as providing multiple charging stations leads to a larger response by consumers. Beyond initial charging infrastructure, we have identified a non-linear relationship between the adoption of emerging EV technology and the size of the associated charging infrastructure network. Our results imply that the greatest effect of incremental infrastructure on EV purchases is when little to no pre-existing infrastructure exists, and when EV sales are already substantial. This is consistent with indirect network effects, and suggests an initial hurdle to the adoption of EVs. Moreover, the response by consumers gradually declines as the pre-existing network infrastructure expands.

Taken together, a low consumer response when existing EV purchases are small and a decreasing marginal installation impact trend can lead to a stand-off between initial EV purchases and charger investments. Once some EVs have been purchased, however, further charger installations do imply indirect network effects, fostering growth in both sides of the market. As the charging network grows, incremental charging infrastructure have a declining impact on EV sales, suggesting declining marginal benefits to consumers. This indicates that unpriced benefits to consumers are largest at the initial stage of the market, suggesting that early government interventions such as subsidies for charging infrastructure deployment have the largest impact on market inefficiencies and EV adoption rate.

Our results further support the view that a behavioral bias magnifies indirect network effects on the market for EVs, as the impact of charging points on EV registrations is consistently lower than that of stations. The fact that consumers respond more to additional installations of charging stations than they do to the addition of more charging points, ceteris paribus, supports the view that consumers’ behavioral response is in part driven by range anxiety. This makes the number of charging points potentially less relevant than the physical presence of a charging station. Furthermore, the evidence that charging stations have a significantly greater effect on EV purchases in urban regions relative to rural or less-urban could indicate potential greater visibility and proximity effects, or hesitancy in rural areas that dampens reactions to new infrastructure. That said, this result further supports our non-linear approach, demonstrating a higher treatment elasticity in municipalities with a larger installed base of chargers (eg. urban, compared to rural).

While our paper contributes to an active research agenda on electric vehicles, we close by emphasizing that much remains to be done. Our analysis does not account for feedback effects from EV purchases to charging station installation, so that our estimate can be seen as a lower bound of the impact of charging infrastructure on EV adoption. Future research may consider how such feedback loops are affected by the pre-existing stock of charging infrastructure.

## Figures and Tables

**Fig. 8:**
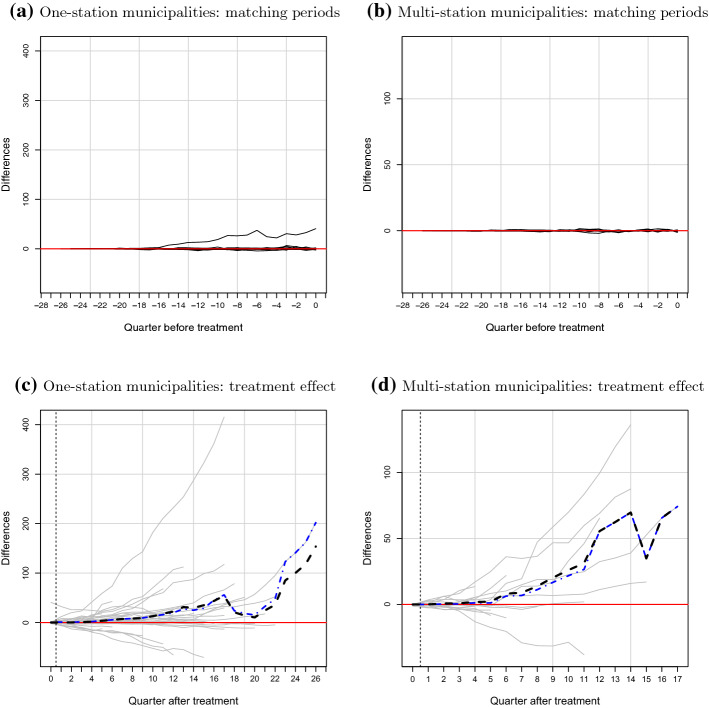
Results from the traditional synthetic control method. *Notes:* The solid gray lines represent the SCM estimated differences between each treated municipality and its synthetic counterpart. The dashed-dotted lines present the mean differences across treated units. The dashed lines provide the mean difference estimated from the ridge-augmented SCM approach reported in Fig. 4

## Appendix 1: Municipalities Used for Synthetic Control Estimation

See Tables 9, 10, and 11.

**Table 9:** Group of one-station municipalities

Municipality code	Municipality name	County	Treatment quarter
135	Råde	Østfold	Q4 2016
227	Fet	Akershus	Q4 2013
239	Hurdal	Akershus	Q4 2015
418	Nord-Odal	Hedmark	Q4 2012
423	Grue	Hedmark	Q4 2014
425	Åsnes	Hedmark	Q4 2013
436	Tolga	Hedmark	Q1 2013
514	Lom	Oppland	Q3 2015
522	Gausdal	Oppland	Q4 2015
536	Søndre Land	Oppland	Q4 2013
619	Ål	Buskerud	Q1 2013
633	Nore og Uvdal	Buskerud	Q3 2012
814	Bamble	Telemark	Q4 2014
817	Drangedal	Telemark	Q4 2015
831	Fyresdal	Telemark	Q1 2017
833	Tokke	Telemark	Q2 2014
937	Evje og Hornnes	Aust-Agder	Q3 2015
1021	Marnardal	Vest-Agder	Q1 2014
1037	Kvinesdal	Vest-Agder	Q3 2013
1114	Bjerkreim	Rogaland	Q4 2016
1121	Time	Rogaland	Q4 2013
1127	Randaberg	Rogaland	Q3 2011
1135	Sauda	Rogaland	Q1 2016
1141	Finnøy	Rogaland	Q3 2015
1142	Rennesøy	Rogaland	Q4 2016
1222	Fitjar	Hordaland	Q4 2013
1231	Ullensvang	Hordaland	Q2 2016
1252	Modalen	Hordaland	Q1 2016
1264	Austrheim	Hordaland	Q2 2013
1417	Vik	Sogn og Fjordane	Q4 2016
1426	Luster	Sogn og Fjordane	Q3 2014
1516	Ulstein	Møre og Romsdal	Q2 2015
1535	Vestnes	Møre og Romsdal	Q4 2016
1551	Eide	Møre og Romsdal	Q2 2014
1822	Leirfjord	Nordland	Q3 2016
1828	Nesna	Nordland	Q4 2016
1850	Tysfjord	Nordland	Q4 2016
1860	Vestvågøy	Nordland	Q3 2016
1871	Andøy	Nordland	Q1 2015
1913	Skånland	Troms	Q4 2016
2017	Kvalsund	Finnmark	Q3 2015
2019	Nordkapp	Finnmark	Q2 2014
5014	Frøya	Trøndelag	Q2 2015
5015	Ørland	Trøndelag	Q3 2012
5022	Rennebu	Trøndelag	Q1 2015
5025	Røros	Trøndelag	Q1 2015
5026	Holtålen	Trøndelag	Q1 2013

This table lists all municipalities included in the group of one-station municipalities. These have initially no charging infrastructure, until they installed a single charging station during the treatment quarter. After that, no more charging infrastructure is installed

**Table 10:** Group of multi-station municipalities

Municipality code	Municipality name	County	Treatment quarter
429	Åmot	Hedmark	Q1 2016
432	Rendalen	Hedmark	Q4 2016
515	Vågå	Oppland	Q1 2017
540	Sør-Aurdal	Oppland	Q4 2016
716	Re	Vestfold	Q2 2015
830	Nissedal	Telemark	Q1 2017
938	Bygland	Aust-Agder	Q2 2015
1211	Etne	Hordaland	Q4 2013
1228	Odda	Hordaland	Q3 2014
1422	Lærdal	Sogn og Fjordane	Q3 2016
1515	Herøy	Møre og Romsdal	Q3 2016
1524	Norddal	Møre og Romsdal	Q2 2014
1865	Vågan	Nordland	Q3 2014
1920	Lavangen	Troms	Q1 2017
1924	Målselv	Troms	Q1 2017
1931	Lenvik	Troms	Q1 2015
5011	Hemne	Trøndelag	Q4 2016

This table lists all municipalities included in the group of multi-station municipalities. These have initially no charging infrastructure, until they installed two or more charging station over a period of four consecutive quarters. In the table, treatment quarter refers to the first of the up to four consecutive quarters where charging stations are installed

**Table 11:** Municipalities included in the donor pool

Municipality code	Municipality name	County
121	Rømskog	Østfold
234	Gjerdrum	Akershus
434	Engerdal	Hedmark
441	Os	Hedmark
541	Etnedal	Oppland
621	Sigdal	Buskerud
628	Hurum	Buskerud
632	Rollag	Buskerud
711	Svelvik	Vestfold
811	Siljan	Telemark
822	Sauherad	Telemark
827	Hjartdal	Telemark
912	Vegårshei	Aust-Agder
919	Froland	Aust-Agder
928	Birkenes	Aust-Agder
935	Iveland	Aust-Agder
1027	Audnedal	Vest-Agder
1029	Lindesnes	Vest-Agder
1034	Hægebostad	Vest-Agder
1111	Sokndal	Rogaland
1119	Hå	Rogaland
1129	Forsand	Rogaland
1130	Strand	Rogaland
1133	Hjelmeland	Rogaland
1144	Kvitsøy	Rogaland
1145	Bokn	Rogaland
1151	Utsira	Rogaland
1234	Granvin	Hordaland
1265	Fedje	Hordaland
1418	Balestrand	Sogn og Fjordane
1424	Årdal	Sogn og Fjordane
1428	Askvoll	Sogn og Fjordane
1438	Bremanger	Sogn og Fjordane
1441	Selje	Sogn og Fjordane
1511	Vanylven	Møre og Romsdal
1514	Sande	Møre og Romsdal
1526	Stordal	Møre og Romsdal
1529	Skodje	Møre og Romsdal
1531	Sula	Møre og Romsdal
1534	Haram	Møre og Romsdal
1543	Nesset	Møre og Romsdal
1545	Midsund	Møre og Romsdal
1546	Sandøy	Møre og Romsdal
1547	Aukra	Møre og Romsdal
1548	Fræna	Møre og Romsdal
1567	Rindal	Møre og Romsdal
1576	Aure	Møre og Romsdal
1811	Bindal	Nordland
1812	Sømna	Nordland
1815	Vega	Nordland
1816	Vevelstad	Nordland
1818	Herøy	Nordland
1827	Dønna	Nordland
1834	Lurøy	Nordland
1835	Træna	Nordland
1836	Rødøy	Nordland
1837	Meløy	Nordland
1838	Gildeskål	Nordland
1839	Beiarn	Nordland
1848	Steigen	Nordland
1851	Lødingen	Nordland
1852	Tjeldsund	Nordland
1856	Røst	Nordland
1857	Værøy	Nordland
1859	Flakstad	Nordland
1866	Hadsel	Nordland
1867	Bø	Nordland
1868	Øksnes	Nordland
1874	Moskenes	Nordland
1911	Kvæfjord	Troms
1917	Ibestad	Troms
1919	Gratangen	Troms
1923	Salangen	Troms
1925	Sørreisa	Troms
1926	Dyrøy	Troms
1927	Tranøy	Troms
1928	Torsken	Troms
1929	Berg	Troms
1936	Karlsøy	Troms
1938	Lyngen	Troms
1940	Gáivuotna Kåfjord	Troms
1941	Skjervøy	Troms
1943	Kvænangen	Troms
2002	Vardø	Finnmark
2003	Vadsø	Finnmark
2011	Guovdageaidnu Kautokeino	Finnmark
2014	Loppa	Finnmark
2015	Hasvik	Finnmark
2021	Karasjohka Karasjok	Finnmark
2022	Lebesby	Finnmark
2023	Gamvik	Finnmark
2024	Berlevåg	Finnmark
2025	Deatnu Tana	Finnmark
2027	Unjargga Nesseby	Finnmark
2028	Båtsfjord	Finnmark
5012	Snillfjord	Trøndelag
5013	Hitra	Trøndelag
5019	Roan	Trøndelag
5020	Osen	Trøndelag
5029	Skaun	Trøndelag
5032	Selbu	Trøndelag
5038	Verdal	Trøndelag
5039	Verran	Trøndelag
5040	Namdalseid	Trøndelag
5043	Røyrvik	Trøndelag
5046	Høylandet	Trøndelag
5048	Fosnes	Trøndelag
5049	Flatanger	Trøndelag
5050	Vikna	Trøndelag
5052	Leka	Trøndelag

This table lists all municipalities included in the donor pool. These have no charging infrastructure over the entire observation period

## Appendix 2: Control Function Estimation Supplements

See Tables 12, 13, 14, 15, and 16.

**Table 12:** First-stage results for charging stations and charging points

	Charging stations	Charging points
	(1)	(2)
IV	0.058***	0.111***
	(0.013)	(0.022)
ln(car price)	− 3.01E−12	− 9.54E−13
	(9.06E−12)	(1.58E−11)
ln(income)	0.196	0.189
	(0.388)	(0.643)
ln(income) × time	− 0.004	0.001
	(0.016)	(0.027)
ln(hybrids) × time	0.003**	− 0.0003
	(0.001)	(0.002)
Constant	− 2.206	− 5.958
	(4.575)	(8.603)
N	366,296	366,296
Adjusted within-R$^2$	0.393	0.355

This table reports first stage regression results for 2SLS and CF procedures. In column (1), the dependent variable is ${ln(charging stations)}_{mit}$. In column (2), the dependent variable is ${ln(charging points)}_{mit}$. See Eq. (2) for the definition of the instrumental variable (IV). All specifications include quarter and municipality-model fixed effects. Standard errors clustered at the municipality level reported in parentheses

*, **, *** respectively denote significance at 10%, 5% and 1% levels

**Table 13:** Polynomial forms of robustness checks—charging stations

	No price	2015 parking	No neighbours	Chargers x BEV	Additional controls	Chargers × time	Chargers × urban
	(1)	(2)	(3)	(4)	(5)	(6)	(7)
ln(charging stations)	0.131***	0.155***	0.146***	0.133**	0.130***	–	–
	(0.045)	(0.041)	(0.050)	(0.053)	(0.050)		
ln(charging stations)$^2$	− 0.036	− 0.037	− 0.036	− 0.050	− 0.036	–	–
	(0.032)	(0.032)	(0.032)	(0.038)	(0.031)		
ln(charging stations)$^3$	0.018**	0.019**	0.018**	0.023**	0.018**	–	–
	(0.009)	(0.009)	(0.009)	(0.011)	(0.009)		
ln(charging stations) x BEV	–	–	–	− 0.004	–	–	–
				(0.026)			
ln(charging stations)$^2$ x BEV	–	–	–	0.031	–	–	–
				(0.029)			
ln(charging stations)$^3$ x BEV	–	–	–	− 0.010	–	–	–
				(0.007)			
ln(charging stations) x early	–	–	–	–	–	0.194***	–
						(0.049)	
ln(charging stations)$^2$ x early	–	–	–	–	–	− 0.055**	–
						(0.025)	
ln(charging stations)$^3$ x early)	–	–	–	–	–	0.023***	–
						(0.008)	
ln(charging stations) x late	–	–	–	–	–	0.144***	–
						(0.054)	
ln(charging stations)$^2$ x late	–	–	–	–	–	− 0.034	–
						(0.032)	
ln(charging stations)$^3$ x late	–	–	–	–	–	0.019**	–
						(0.009)	
ln(charging stations) × urban	–	–	–	–	–	–	0.019
							(0.366)
ln(charging stations)$^2$ × urban	–	–	–	–	–	–	− 0.008
							(0.165)
ln(charging stations)$^3$ × urban	–	–	–	–	–	–	0.015
							(0.028)
ln(charging stations) x town	–	–	–	–	–	–	0.182***
							(0.063)
ln(charging stations)$^2$ x town	–	–	–	–	–	–	− 0.091*
							(0.050)
ln(charging stations)$^3$ x town	–	–	–	–	–	–	0.034**
							(0.013)
ln(charging stations) x rural	–	–	–	–	–	–	0.168***
							(0.039)
ln(charging stations)$^2$ x rural	–	–	–	–	–	–	− 0.074***
							(0.025)
ln(charging stations)$^3$ x rural	–	–	–	–	–	–	0.027***
							(0.008)
ln(car price)	–	0.110***	0.110***	0.109***	0.110***	0.110***	0.110***
		(0.008)	(0.009)	(0.009)	(0.009)	(0.008)	(0.009)
ln(income)	− 0.130	− 0.136*	− 0.134	− 0.130	− 0.122	− 0.136*	− 0.163**
	(0.077)	(0.092)	(0.078)	(0.084)	(0.073)	(0.075)	(0.082)
ln(income) × time	0.003	0.003	0.003	0.003	0.002	0.003	0.004
	(0.004)	(0.004)	(0.004)	(0.004)	(0.004)	(0.004)	(0.004)
ln(hybrids) × time	0.006***	0.006***	0.006***	0.006***	0.006***	0.006***	0.006***
	(0.001)	(0.001)	(0.001)	(0.001)	(0.001)	(0.001)	(0.001)
ln(population)	–	–	–	–	− 0.082	–	–
					(0.096)		
Proportion of detached and duplex dwellings	–	–	–	–	0.001	–	–
					(0.002)		
First stage residual	− 0.136***	− 0.158***	− 0.150***	− 0.135***	− 0.134***	− 0.160***	− 0.138***
	(0.033)	(0.037)	(0.038)	(0.035)	(0.041)	(0.047)	(0.037)
Constant	0.360	− 1.081	− 1.075	− 1.044	− 1.775	− 1.238	− 1.243
	(1.052)	(1.067)	(0.902)	(0.965)	(1.184)	(1.150)	(1.161)
N	366,296	366,296	366,296	366,296	366,296	366,296	366,296
Adjusted within-R$^2$	0.0806	0.0817	0.0817	0.0820	0.0817	0.0821	0.0820
1st-stage partial F-stat.	18.32	11.29	16.80	19.01	19.51	19.01	19.01

In all columns, the dependent variable is the log of new electric vehicle registrations (${ln(EV)}_{mit}$). Column (1) omits the car price variable. Column (2) uses the number of parking spaces in 2015 to construct the instrument. Column (3) excludes neighboring municipalities to construct the instrument. In column (4), we interact the treatment variable with a dummy for battery-only EVs. Column (5) includes further control variables. In column (6), we estimate separate elasticities for observations in 2010–2013 and 2014–2017. Column (7) estimates separate elasticities by municipal degree of urbanisation—urban/city, town/suburban, rural. All specifications are estimated with a control function approach and include quarter and municipality-model fixed effects. The 1st stage partial F-statistic for the instrumental variable is derived from first-stage regression results. Standard errors bootstrapped with 500 replications and clustered at the municipality level reported in parentheses

*, **, and *** respectively denote significance at 10%, 5% and 1% levels

**Table 14:** Polynomial forms of robustness checks—charging points

	No price	2015 parking	No neighbours	Chargers x BEV	Additional controls	Chargers × time	Chargers × urban
	(1)	(2)	(3)	(4)	(5)	(6)	(7)
ln(charging points)	0.132***	0.153***	0.136***	0.152***	0.131***	–	–
	(0.020)	(0.023)	(0.021)	(0.017)	(0.020)		
ln(charging points)$^2$	− 0.055***	− 0.056***	− 0.055***	− 0.073***	− 0.055***	–	–
	(0.008)	(0.009)	(0.009)	(0.009)	(0.008)		
ln(charging points)$^3$	0.012***	0.012***	0.012***	0.015***	0.012***	–	–
	(0.002)	(0.002)	(0.002)	(0.002)	(0.002)		
ln(charging points) x BEV	–	–	–	− 0.048***	–	–	–
				(0.016)			
ln(charging points)$^2$ x BEV	–	–	–	0.041***	–	–	–
				(0.011)			
ln(charging points)$^3$ x BEV	–	–	–	− 0.007***	–	–	–
				(0.002)			
ln(charging points) x early	–	–	–	–	–	0.147***	–
						(0.026)	
ln(charging points)$^2$ x early	–	–	–	–	–	− 0.053***	–
						(0.018)	
ln(charging points)$^3$ x early	–	–	–	–	–	0.012***	–
						(0.003)	
ln(charging points) x late	–	–	–	–	–	0.134***	–
						(0.019)	
ln(charging points)$^2$ x late	–	–	–	–	–	− 0.054***	–
						(0.011)	
ln(charging points)$^3$ x late	–	–	–	–	–	0.012***	–
						(0.002)	
ln(charging points) × urban	–	–	–	–	–	–	0.080
							(1.045)
ln(charging points)$^2$ × urban	–	–	–	–	–	–	− 0.041
							(0.243)
ln(charging points)$^3$ × urban	–	–	–	–	–	–	0.011
							(0.019)
ln(charging points) x town	–	–	–	–	–	–	0.149***
							(0.031)
ln(charging points)$^2$ x town	–	–	–	–	–	–	− 0.068***
							(0.018)
ln(charging points)$^3$ x town	–	–	–	–	–	–	0.015***
							(0.003)
ln(charging points) x rural	–	–	–	–	–	–	0.102***
							(0.021)
ln(charging points)$^2$ x rural	–	–	–	–	–	–	− 0.028**
							(0.013)
ln(charging points)$^3$ x rural	–	–	–	–	–	–	0.006**
							(0.003)
ln(car price)	–	0.110***	0.110***	0.109***	0.110***	0.110***	0.110***
		(0.008)	(0.008)	(0.006)	(0.008)	(0.010)	(0.007)
ln(income)	− 0.108	− 0.113	− 0.109	− 0.107	− 0.105	− 0.112	− 0.119
	(0.070)	(0.066)	(0.069)	(0.099)	(0.067)	(0.082)	(0.083)
ln(income) × time	0.002	0.002	0.002	0.002	0.002	0.003	0.003
	(0.004)	(0.003)	(0.004)	(0.004)	(0.004)	(0.004)	(0.004)
ln(hybrids) × time	0.006***	0.006***	0.006***	0.006***	0.006***	0.006***	0.006***
	(0.0005)	(0.001)	(0.0004)	(0.0004)	(0.001)	(0.0004)	(0.001)
ln(population)	–	–	–	–	0.057	–	–
					(0.098)		
Proportion of detached and duplex dwellings	–	–	–	–	0.001	–	–
					(0.002)		
First stage residual	− 0.079***	− 0.099***	− 0.083***	− 0.079***	− 0.079***	− 0.088***	− 0.078***
	(0.018)	(0.023)	(0.018)	(0.017)	(0.015)	(0.017)	(0.016)
Constant	0.303	− 1.142	− 1.123	− 1.112	− 1.651	− 1.347	− 1.108
	(1.017)	(0.811)	(1.145)	(0.946)	(1.411)	(1.014)	(1.072)
N	366,296	366,296	366,296	366,296	366,296	366,296	366,296
Adjusted within-R$^2$	0.0815	0.0826	0.0826	0.0830	0.0826	0.0828	0.0830
1st-stage partial F-stat.	24.57	14.17	23.73	25.54	23.04	25.54	25.54

In all columns, the dependent variable is the log of new electric vehicle registrations (${ln(EV)}_{mit}$). Column (1) omits the car price variable. Column (2) uses the number of parking spaces in 2015 to construct the instrument. Column (3) excludes neighboring municipalities to construct the instrument. In column (4), we interact the treatment variable with a dummy for battery-only EVs. Column (5) includes further control variables. In column (6), we estimate separate elasticities for observations in 2010–2013 and 2014–2017. Column (7) estimates separate elasticities by municipal degree of urbanisation—urban/city, town/suburban, rural. All specifications are estimated with a control function approach and include quarter and municipality-model fixed effects. The 1st stage partial F-statistic for the instrumental variable is derived from first-stage regression results. Standard errors bootstrapped with 500 replications and clustered at the municipality level reported in parentheses

*, **, and *** respectively denote significance at 10%, 5% and 1% levels

**Table 15:** First-stage results for robustness checks—charging stations

	No price	2015 parking	No neighbours	Chargers x BEV	Additional controls	Chargers × time	Chargers × urban
	(1)	(2)	(3)	(4)	(5)	(6)	(7)
IV	0.058***	–	–	0.058***	0.057***	0.058***	0.058***
	(0.014)			(0.013)	(0.013)	(0.013)	(0.013)
IV-parking 2015	–	0.049***	–	–	–	–	–
		(0.015)					
IV-no neighbors	–	–	0.055***	–	–	–	–
			(0.014)				
ln(car price)	–	-2.05E−12	-3.17E−12	-3.01E−12	− 5.64E−12	-3.01E−12	-3.01E−12
		(8.76E−12)	(8.75E−12)	(9.06E−12)	(9.09E−12)	(9.06E−12)	(9.06E−12)
ln(income)	0.196	0.170	0.205	0.196	0.307	0.196	0.196
	(0.374)	(0.396)	(0.373)	(0.388)	(0.402)	(0.388)	(0.388)
ln(income) × time	− 0.004	− 0.002	− 0.004	− 0.004	− 0.008	− 0.004	− 0.004
	(0.016)	(0.015)	(0.015)	(0.016)	(0.016)	(0.016)	(0.016)
ln(hybrids) × time	0.003**	0.003**	0.003**	0.003**	0.003**	0.003**	0.003**
	(0.001)	(0.001)	(0.001)	(0.001)	(0.001)	(0.001)	(0.001)
ln(population)	–	–	–	–	0.446***	–	–
					(0.586)		
Proportion of detached and duplex dwellings	–	–	–	–	− 0.003	–	–
					(0.009)		
Constant	-2.206	-2.231	-2.076	-2.206	− 5.280	-2.206	-2.206
	(4.270)	(4.398)	(4.263)	(4.575)	(6.324)	(4.575)	(4.575)
N	366,296	366,296	366,296	366,296	366,296	366,296	366,296
Adjusted within-R$^2$	0.393	0.390	0.392	0.393	0.394	0.393	0.393

This table reports first stage regression results for robustness checks. In all columns, the dependent variable is ${ln(charging stations)}_{mit}$. See Eq. (2) for the definition of the instrumental variable (IV). All specifications include quarter and municipality-model fixed effects. Standard errors clustered at the municipality level reported in parentheses

*, **, and *** respectively denote significance at 10%, 5% and 1% levels

**Table 16:** First-stage results for robustness checks—charging points

	No price	2015 parking	No neighbours	Chargers x BEV	Additional controls	Chargers × time	Chargers × urban
	(1)	(2)	(3)	(4)	(5)	(6)	(7)
IV	0.111***	–	–	0.110***	0.108***	0.110***	0.110***
	(0.022)			(0.022)	(0.023)	(0.022)	(0.022)
IV-parking 2015	–	0.089***	–	–	–	–	–
		(0.024)					
IV-no neighbours	–	–	0.109***	–	–	–	–
			(0.020)				
ln(car price)	–	5.70E−13	− 1.53E−12	1.18E−12	− 6.45E−12	1.18E−12	1.18E−12
		(1.67E−11)	(1.60E−11)	(1.03E−11)	(1.73E−11)	(1.03E−11)	(1.03E−11)
ln(income)	0.189	0.135	0.208	0.134	0.425	0.134	0.134
	(0.688)	(0.729)	(0.671)	(0.646)	(0.775)	(0.646)	(0.646)
ln(income) × time	0.001	0.004	0.0004	0.006	− 0.008	0.006	0.006
	(0.029)	(0.029)	(0.028)	(0.028)	(0.030)	(0.028)	(0.028)
ln(hybrids) × time	− 0.0003	0.0004	− 0.0003	− 0.001	− 0.001	− 0.001	− 0.001
	(0.002)	(0.002)	(0.028)	(0.002)	(0.002)	(0.002)	(0.002)
ln(population)	–	–	–	–	0.882*	–	–
					(0.1.074)		
Proportion of detached and duplex dwellings	–	–	–	–	− 0.008	–	–
					(0.030)		
Constant	− 5.958	− 5.494	− 5.731	− 5.438	-11.824	− 5.438	− 5.438
	(8.391)	(9.238)	(8.567)	(7.620)	(12.546)	(7.620)	(7.620)
N	366,296	366,296	366,296	366,296	366,296	366,296	366,296
Adjusted within-R$^2$	0.355	0.349	0.354	0.355	0.355	0.355	0.355

This table reports first stage regression results for robustness checks. In all columns, the dependent variable is ${ln(charging points)}_{mit}$. See Eq. (2) for the definition of the instrumental variable (IV). All specifications include quarter and municipality-model fixed effects. Standard errors clustered at the municipality level reported in parentheses

*, **, and *** respectively denote significance at 10%, 5% and 1% levels

## Appendix 3: Synthetic Control Method Supplements

As outlined in Sect. 2.3, the ridge-augmented SCM from Ben-Michael et al. (2018) offers an improvement in SCM case study analysis by allowing for a more precise matching and hence lower MSPE. In Fig. 8, we compare a traditional SCM matching algorithm (Abadie and Gardeazabal 2003; Abadie et al. 2010) to the ridge-augmented SCM results presented in the main text.

Results suggest that the pre-treatment residuals (Fig. 8a, b) are significantly larger and display more variability as compared to our main results. This lower fit of the synthetic municipalities confirms that the ridge-augmented SCM approach provides a more accurate estimate of the counterfactual, and in turn the treatment effects.

Nevertheless, Fig. 8c, d show that the estimated post-treatment differences are qualitatively similar using both approach. As expected, larger MPSE implies additional variability in early post-treatment quarters. However, overall, the average treatment effect is very similar with both approaches. This is also illustrated in Table 17, which provides the mean and median treatment effect for each quarter associated with a traditional SCM (Tables 18, 19, and 20).

**Table 17:** Summary results for the traditional synthetic control method

Quarter post-treatment	One-station municipalities			Multi-station municipalities		
	Obs.	Mean	Median	Obs.	Mean	Median
1	47	0.87	− 0.06	17	0.04	− 0.08
2	47	0.84	0.04	17	0.28	− 0.27
3	47	1.14	0.50	17	0.35	− 0.10
4	47	1.89	0.52	17	1.19	− 0.58
5	46	3.68	0.86	13	1.08	− 0.93
6	38	5.42	2.64	10	6.28	6.44
7	36	7.19	4.16	8	6.76	10.05
8	35	8.14	4.02	8	11.17	10.52
9	33	9.24	5.64	7	16.68	16.70
10	30	12.79	7.07	7	21.71	22.60
11	26	15.96	6.61	7	26.52	24.80
12	24	19.67	7.40	5	55.52	65.40
13	20	27.57	8.55	4	62.39	58.27
14	18	24.88	6.26	4	69.67	63.25
15	17	28.89	4.98	2	34.80	34.80
16	14	45.15	18.62	1	65.60	65.60
17	13	55.89	29.49	1	74.20	74.20
18	8	23.84	20.11			
19	7	18.28	11.76			
20	6	15.28	5.88			
21	4	32.51	30.62			
22	3	47.48	52.23			
23	1	123.00	123.00			
24	1	141.00	141.00			
25	1	162.00	162.00			
26	1	202.00	202.00			

This table summarizes results for the post-treatment gap in cumulative EV stock between treated municipalities and synthetic controls. Mean and median reported refer to the distribution of treatment effects estimated from the traditional SCM

**Table 18:** Summary results for the temporal placebo tests

Quarter post-treatment	One-station municipalities			Multi-station municipalities		
	Obs.	Mean	Median	Obs.	Mean	Median
1	46	0.61	− 0.06	16	0.47	0.10
2	46	0.69	− 0.04	16	0.17	− 0.18
3	46	0.54	− 0.10	16	0.99	1.03
4	46	0.78	− 0.13	16	1.08	1.31
5	46	0.83	0.18	16	1.31	1.67
6	46	0.68	0.52	16	2.03	1.81
7	46	1.50	0.48	16	2.85	1.93
8	46	2.22	0.48	16	4.69	2.59
9	45	4.09	0.89	12	4.59	2.65
10	37	8.69	2.34	9	11.94	8.86
11	35	11.18	7.57	7	14.19	16.22
12	34	12.83	6.55	7	19.18	16.10
13	32	15.40	9.18	6	24.80	19.32
14	29	18.88	4.50	6	29.81	28.98
15	25	20.64	13.05	6	36.77	36.16
16	23	28.24	21.22	5	54.30	63.63
17	19	31.63	21.89	4	60.49	59.13
18	17	29.05	4.67	4	66.55	63.09
19	16	32.85	4.63	2	30.39	30.39
20	13	45.06	10.50	1	65.80	65.80
21	12	53.21	8.97	1	74.45	74.45
22	7	20.05	5.67			
23	6	8.88	2.18			
24	5	2.36	− 6.30			
25	3	16.17	13.58			
26	2	22.11	22.11			

This table summarizes results for the post-treatment gap in cumulative EV stock for non-treated municipalities subject to placebo treatments 4-quarter before treatment, and synthetic controls. We report mean and median placebo treatment effects

**Table 19:** City-proximate donor pool municipalities

Municipality code	Municipality name	County
234	Gjerdrum	Akershus
711	Svelvik	Vestfold
1119	Hå	Rogaland
1129	Forsand	Rogaland
1130	Strand	Rogaland
5029	Skaun	Trøndelag

This table lists all municipalities included in the donor pool that have been calculated to have a one hour or less driving time from their population-weighted geographical centre to that of a city or urban municipality

**Table 20:** Summary of post-treatment synthetic control results under “leave-one-out” test

Quarter post-treatment	One-station municipalities			Multi-station municipalities		
	Obs.	Mean	Median	Obs.	Mean	Median
1	47	− 0.34	− 0.15	17	0.00	− 0.01
2	47	− 0.30	− 0.09	17	0.36	− 0.05
3	47	0.10	0.36	17	0.50	− 0.10
4	47	0.88	0.52	17	1.49	− 0.14
5	46	2.57	0.71	13	1.62	− 0.17
6	38	3.61	1.56	10	7.20	6.08
7	36	5.27	4.22	8	8.00	9.38
8	35	5.74	3.61	8	12.88	9.63
9	33	7.29	5.64	7	18.55	14.28
10	30	10.26	4.94	7	24.03	22.60
11	26	14.92	6.63	7	29.58	24.80
12	24	19.15	10.71	5	54.68	60.80
13	20	27.76	14.90	4	62.61	60.89
14	18	25.17	12.83	4	69.51	66.17
15	17	29.08	12.91	2	34.80	34.80
16	14	39.69	18.75	1	65.60	65.60
17	13	48.35	29.78	1	74.20	74.20
18	8	39.62	20.11			
19	7	38.67	11.76			
20	6	39.94	5.88			
21	4	71.59	30.62			
22	3	103.16	52.23			
23	1	298.83	298.83			
24	1	316.45	316.45			
25	1	356.75	356.75			
26	1	414.40	414.40			

This table summarizes results for the post-treatment gap in cumulative EV stock between treated municipalities and synthetic controls under the “leave-one-out” test. Mean and median reported refer to the distribution of treatment effects estimated from the ridge-augmented SCM

## Footnotes

1 The transport sector is responsible for about 25% of GHG emissions globally (International Energy Agency 2019), 57% of Nitrous Oxides, and 20% of particulate matter 2.5 (European Environmental Agency 2018). See International Energy Agency (2019) for projected trends in EV adoption. Importantly, Holland et al. (2016) emphasize regional heterogeneity in the benefits associated with the electrification of transports in relation to the use of alternative electricity generation technologies. Norway produces around 98% of its electricity from hydro and wind sources (SSB 2018).

2 Note Bjerkan et al. (2016) did not address charging stations.

3 In this we include all registered public parking spaces (SSB 2018) such as government-controlled on- and off-street parking, schools, churches, sports facilities, and other parking lots.

4 Not all charging stations must be on public parking places, especially fast chargers, however, we exploit the correlation between the two, given the existing use of public spaces. Li et al. (2017), for example, show the use of private (grocery store) parking spaces, however in Norway these data are lacking.

5 This approach is based on Hausman (1978) and Heckman and Robb (1985), as described by Wooldridge (2015). It is similar to Terza et al. (2008)’s otherwise named two-stage residual inclusion procedure.

6 We note, however, that our results do not account for second-order feedback effects from EV purchases to charging station installation. These elasticities can therefore be interpreted as lower bound estimates.

7 The average daily distance travelled in Norway is around 47 km (Hjorthol et al. 2014).

8 The SCM is a quantitative tool for case study analysis which can be applied in situations where there is no clear, observed counterfactual for comparison. See for example Moser (2005), Mideksa (2013), Barone and Mocetti (2014), Andersson (2019) and Clinton and Steinberg (2019).

9 A related literature studies the adoption of alternative-fuel vehicles and the provision of fueling infrastructure. For example, Corts (2010) and Shriver (2015) provide empirical evidence that fueling stations supplying ethanol increase the adoption of ethanol-compatible vehicles in the U.S., and discuss the provision of subsidies to fuel retailers.

10 On the impact of direct financial incentives on EV and hybrid purchases, see also DeShazo et al. (2017), Sallee (2011), Beresteanu and Li (2011), Chandra et al. (2010), Gallagher and Muehlegger (2011), and Diamond (2009).

11 While the purpose of our work is different, our data is closely related to Springel (2021), with a few differences. First, we work at a more disaggregated municipality-level, with 422 cross-sectional units instead of 19 counties. Second, our analysis includes 2016 and 2017, and during 3 years EV sales increased by more than 80%, charging stations rose by a quarter, and charging points grew by around 40% (see Fig. 1). Lastly, our data covers both charging stations and charging points.

12 Note that we do not differentiate chargers by speed, connector type, owning company, or access and usage fees. This includes both so-called ‘fast’ (level 3) and ‘slow’ (level 2) chargers, the majority of which (85% on average) are the latter (see Li (2019) for a discussion of charger types and compatibility, and Greaker (2021) on fast charger standardisation).

13 The NOBIL database is only available from 2010 and thus limits our panel’s time range.

14 “Appendix 1” lists the names of these 3 groups of municipalities, and provides the quarters of charger installation.

15 We deal with values of zero EVs, charging stations/points, and parking places by adding one before log-transforming the data. A comparison to conducting an inverse hyperbolic sine transformation (Bellemare and Wichman 2020) generates no significant difference in estimated coefficients.

16 Note that this specification is very similar to structural models for market shares (Berry 1994), although for our purpose a linear specification makes the elasticity interpretation more transparent. See Li et al. (2017) for further discussion of this issue.

17 Bootstrapped standard errors are estimated based on 500 replications.

18 As discussed in Berry (1994) and Berry et al. (1995), the price variable is likely endogenous because of unobserved quality attributes. In our setting, however, our focus is on identification of the coefficient associated with charging infrastructure, and our IV strategy implies that we do not necessarily need to control for car prices.

19 Note that, ideally, we would want to consider parking space data before 2010, but 2015 is the first year for statistics on parking spaces were collected.

20 See also Abadie (2021) for a discussion of SCM extensions and bias-correction methods.

21 Following Andersson (2019), we restrict the analysis of placebo results to municipalities in the donor pool for which a good synthetic unit can be found. In particular, we consider only those units with a MSPE smaller or equal to the worse fit achieved in our set of treated units. This focuses the comparison among units for which the fit of the SCM approach is similar.

22 Note that results in column (1) and (4) correspond to Table 3, column (2) and (4) respectively, illustrating that 2SLS and CF procedures generate the same coefficient estimates whereas bootstrapped standard errors differ slightly. First-stage results remain the same and are reported in “Appendix 2” Table 12.

23 It suffices to note here that robustness results for the cubic specifications do not substantially differ from the primary results in Table 4, and that explanations for linear robustness checks in Tables 5 and 6 apply to the cubic specifications, too.

24 As expected, the ridge augmented SCM provides a more precise matching relative to the traditional SCM, as shown in Fig 8a, b. Treatment effects, discussed next, are however consistent across the two approaches.

25 Due to the differing dates of charging station installation across treated units, the number of post-treatment periods varies across municipalities. One implication, shown in Table 7, is that the number of treated-synthetic municipality pairs declines over time from the initial provision of charging infrastructure.

26 Placebo tests include only estimates for which pre-treatment MSPE is at least as good as the largest MPSE obtained for treated units. This leads us to exclude 7 municipalities from the one-station group (out of 2200) and 134 municipalities from the multi-station group (out of 990).

27 The p-value for the average treatment effect estimate is also below 10% after the 23rd quarter, although it refers to only one municipality. See Table 7.

28 Specifically, we calculate the driving time on a representative weekday morning between the population-weighted geographical centre-points of each donor and urban municipality using the *georoute* Stata package (Weber and Péclat 2017).

## Declarations

### Statement of exclusive submission

This paper has not been submitted elsewhere in identical form, nor will it be during the first three months after its submission to the Publisher.
